# Butyrate-producing gut bacteria restrain PBAT microplastic-triggered brain microglial lipotoxicity via a microbiota–butyrate–mTORC1–ISR relay along the gut–brain axis

**DOI:** 10.1186/s12974-026-03869-1

**Published:** 2026-05-13

**Authors:** Ming-Zhu Wang, Ze-Bang Du, Wen-Qi Xu, Yu-Han Xie, Lei-Lei Wang, Xin-Xin He, Yu-Han Wang, Han-Ying Zheng, You-Liang Yao, Ya-Bin Song, Zhong-Ning Lin, Yu-Chun Lin

**Affiliations:** 1 State Key Laboratory of Vaccines for Infectious Diseases, Xiang An Biomedicine Laboratory, National Innovation Platform for Industry-Education Integration in Vaccine Research, School of Public Health, Xiang’an Hospital of Xiamen University, Xiamen University, Xiamen, 361102 China; 2 Fujian Medical University, Fuzhou, Fujian, China; 3 Department of Neurology, Xiang’an Hospital of Xiamen University, Xiamen, Fujian China

**Keywords:** PBAT microplastics, Gut–brain axis, Butyrate, Microglial lipotoxicity, mTORC1–ISR signaling

## Abstract

Eco-friendly poly(butylene adipate-co-terephthalate) (PBAT) is widely marketed as biodegradable, yet the neurotoxicity of derived PBAT microplastics (PBAT-MPs) and their underlying mechanisms remain poorly characterized. Here we identify a previously unrecognized “gut microbiota–butyrate–neuro-lipid” axis linking intestinal PBAT-MPs exposure to hippocampal microglial lipotoxicity and cognitive impairment. By integrating fecal microbiota transplantation (FMT) with multi-omics analyses, we demonstrate that orally administered PBAT-MPs preferentially accumulate in the colon, impair epithelial barrier integrity, deplete butyrate-associated taxa, including *Muribaculaceae* and *Alloprevotella*, and enrich *Escherichia–Shigella*. Butyrate depletion elevates systemic lipopolysaccharide (LPS) levels and, via the gut–brain inflammatory route, activates mTORC1–integrated stress response (ISR) signaling in microglia. Consequently, microglia acquire a lipotoxic phenotype characterized by transcriptional up-regulation of *DGAT*- and *ACSL*-dependent lipid droplet (LD) biogenesis genes, accumulation of toxic lipids and inflammatory mediators, synaptic stripping, and memory loss. In vivo butyrate supplementation in PBAT-MP-exposed mice alleviates hippocampal pathology, normalizes microglial lipid accumulation, suppresses neuroinflammation, reduces ceramide levels, and improves cognitive performance. Mechanistically, butyrate inhibits mTORC1, attenuates eIF2α–ATF4-dependent ISR signaling, and represses DGAT/ACSL-dependent LD biogenesis, whereas microglial Rptor overexpression abolishes these protective effects, identifying mTORC1 as an upstream metabolic checkpoint. Collectively, our findings establish the microbiota–butyrate–mTORC1–ISR relay as a core driver of PBAT-MPs-induced neurotoxicity and highlight restoration of butyrate signaling as a promising microbiota-based strategy for preventing microplastic-induced brain lipotoxic injury.

**Graphical Abstract:**

**Figure:**
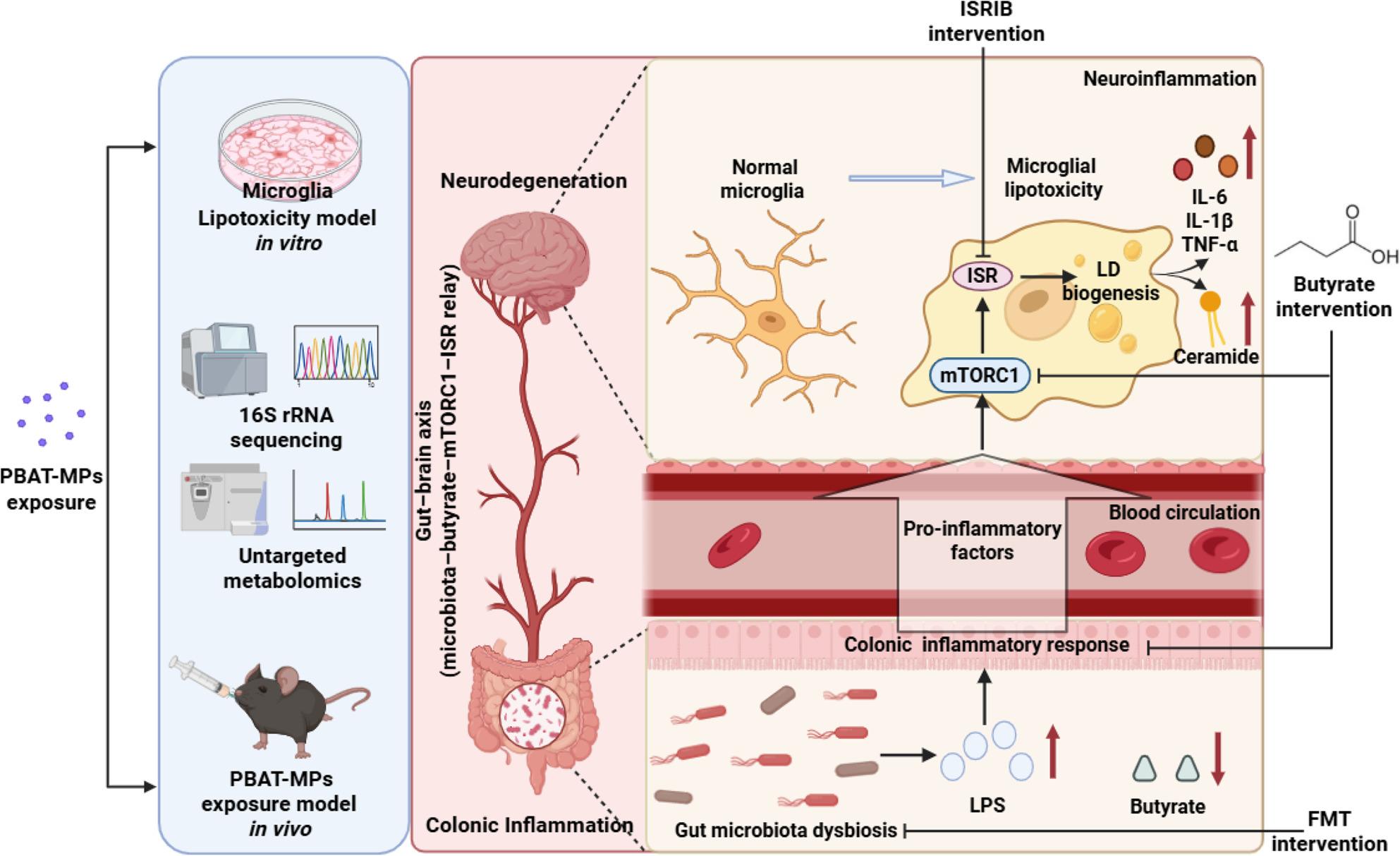
Proposed molecular mechanism underlying poly(butylene adipate-co-terephthalate) microplastics (PBAT-MPs) neurotoxicity via gut–brain immunoinflammatory axis: PBAT-MPs exposure disrupts gut microbial homeostasis, depletes the protective metabolite butyrate, and activates microglial mTORC1–integrated stress response (ISR) signaling, thereby driving lipid-droplet (LD) biogenesis. These events culminate in lipotoxicity-associated neuroinflammation and neurodegenerative injury. (Created with BioRender.com).

**Supplementary Information:**

The online version contains supplementary material available at 10.1186/s12974-026-03869-1.

## Introduction

The escalating global plastic pollution crisis has accelerated the development and adoption of biodegradable plastics (BPs) as sustainable alternatives to conventional petroleum-based plastics [1]. Among these, poly(butylene adipate-co-terephthalate) (PBAT) has emerged as a widely used biodegradable polymer because of its favorable mechanical properties and broad applications in food packaging, agriculture, electronics, and medical devices [2]. However, growing evidence has challenged the environmental benefits initially attributed to PBAT [3]. Although PBAT can degrade efficiently under controlled industrial composting conditions, achieving complete mineralization within ~3 months [4], its fate in natural environments is markedly different. Recent studies suggest that PBAT can persist for up to 3 years under ambient conditions [5]. Moreover, its biodegradable nature accelerates fragmentation relative to conventional plastics, thereby increasing microplastic (MP) generation [6]. This process produces substantial quantities of PBAT microplastics (PBAT-MPs), raising concerns regarding ecological and health risks. Emerging evidence indicates that PBAT-MPs exposure induces multi-organ toxicity in aquatic species, including zebrafish and invertebrates [7]. Notably, sub-chronic oral exposure (21 days) to environmentally relevant concentrations of PBAT-MPs triggers behavioral abnormalities and anxiety-like phenotypes in zebrafish, accompanied by activation of immune-related pathways in the brain [8]. Together, these findings suggest that PBAT-MPs may exert neurotoxic effects through neuroimmune mechanisms and may contribute to neurodegenerative processes.

Oral ingestion is considered the predominant route of human exposure to biodegradable microplastics (BMPs), positioning the gastrointestinal (GI) tract as the first biological barrier and early target of toxicity [9]. Following ingestion, PBAT-MPs accumulate preferentially in intestinal mucosa, where they induce gut dysbiosis characterized by reduced microbial diversity and altered metabolite profiles [10]. According to the gut–brain axis framework, such microbial disturbances may influence the central nervous system (CNS) through interconnected pathways involving neurotransmitter regulation, endocrine signaling, and systemic/neuroinflammation [11]. As the largest immune organ, intestinal mucosa rapidly senses exogenous particles and can transmit inflammatory signals to the brain via vagal and cytokine-mediated pathways [12]. Whether PBAT-MPs promote this gut–brain inflammatory relay to precipitate neurodegeneration remains untested. We therefore hypothesized that PBAT-MPs induce neurotoxicity indirectly by disrupting gut microbial homeostasis and activating a gut–brain inflammatory cascade. Defining this mechanism is essential for accurate risk assessment of BMPs and for developing gut-targeted strategies to mitigate potential neurological effects.

Single-cell profiling has identified microglia as key sentinel cells of the gut–brain axis that respond rapidly to intestinal inflammatory cues [13]. As resident CNS macrophages, microglia not only survey parenchyma but also shape neuronal circuits through synaptic pruning and modulation of neural plasticity [14]. Recent evidence indicates that chronic peripheral inflammation can reprogramme microglia toward a lipotoxic state characterized by excessive lipid droplet (LD) accumulation, a ceramide-rich secretory profile, and sustained pro-inflammatory signaling [15, 16]. Importantly, genetic or pharmacological LD clearance reverses this phenotype and alleviates associated neurodegeneration [17, 18], highlighting microglial lipotoxicity as a potential therapeutic target in inflammation-driven neuronal injury. We therefore hypothesized that PBAT-MPs activate a gut-to-brain inflammatory cascade that induces a lipotoxic microglial phenotype, thereby promoting neurodegeneration. Clarifying this mechanism may define how BMPs impair brain health at the cellular level and identify lipid-centered intervention strategies.

The integrated stress response (ISR), defined by stress-triggered phosphorylation of eukaryotic initiation factor 2α (eIF2α) and subsequent translational activation of transcription factor ATF4, is a conserved adaptive pathway that suppresses global protein synthesis whilst reshaping cellular metabolism [19]. In microglia, persistent ISR activation disrupts lipid homeostasis by inducing lipogenic enzymes, including FASN, DGAT, and ACSL family members, thereby promoting LDs accumulation and ceramide-driven neurotoxicity [20, 21]. Conversely, mammalian target of rapamycin complex 1 (mTORC1), a nutrient-sensing pathway influenced by gut microbiota-derived short-chain fatty acids (SCFAs), regulates anabolic lipid metabolism and has recently been implicated in *ATF4* transcriptional control [22]. These findings suggest a potential mechanistic intersection between mTORC1 and ISR. Whether PBAT-MPs-induced SCFA depletion drives microglial lipotoxicity through the mTORC1–ISR axis remains unknown. Addressing this question may clarify how gut microbial metabolites remotely regulate microglial lipid metabolism and may reveal precise targets for mitigating BMP-induced neurotoxicity.

Here, we test the hypothesis that PBAT-MPs trigger a gut–mTORC1–ISR axis that drives microglial LD biogenesis and lipotoxicity, thereby contributing to neurodegeneration. Defining this gut–brain lipid signaling pathway may reveal actionable therapeutic targets, including SCFA-dependent mTORC1 modulation and selective ISR/ATF4 pathway inhibition, for early prevention of BMP-associated neurodegenerative disorders.

## Materials and methods

### Chemicals and reagents

Red-fluorescent PBAT-MPs (1–2 μm, λex/em 632/680 nm, coefficient of variation < 5%) were obtained from Zhongke Keyou Technology Co., Ltd. (Beijing, China). DAPI and Hoechst 33342 were purchased from Beyotime (Shanghai, China). Integrated stress response inhibitor (ISRIB), lipopolysaccharide (LPS, *E. coli* O55:B5), and sodium butyrate (≥ 98% purity) were purchased from Sigma-Aldrich (St. Louis, MO, USA). BODIPY™ 493/503 was purchased from Thermo Fisher Scientific (Waltham, MA, USA). ELISA kits for mouse and human IL-6, IL-1β, TNF-α, LPS, ceramide, and butyrate were purchased from ABclonal (Wuhan, Hubei, China), Yaji Biotechnology Co., Ltd. (Shanghai, China), and ELK Biotechnology Co., Ltd. (Wuhan, Hubei, China), respectively.

### Characterization of PBAT-MPs in an in vitro simulated intestinal digestion model

Intestinal microorganisms were collected as described previously [23]. The microbial pellet was resuspended in modified GAM medium (Solarbio Science & Technology Co., Ltd., Beijing, China) and adjusted to OD_600_ = 1.0. Subsequently, 1 g PBAT-MPs was added to 30 mL microbial suspension, whilst GAM medium alone served as control. Cultures were incubated at 37 °C in a fermenter (BIOFLO 120, Eppendorf, Hamburg, Germany) with continuous agitation at 50 rpm. Medium was replaced every 3 days, and microbial density was readjusted to OD_600_ = 1.0 at each exchange. Samples were collected every 2 weeks for analysis. PBAT-MP morphology was examined using focused-ion-beam scanning electron microscope (Helios 5 UC, Thermo Fisher Scientific). Degradation was assessed by gravimetric analysis. Hydrodynamic diameter and zeta potential were measured in ultrapure water and simulated intestinal fluid (pH 6.8) at 25 °C using dynamic light scattering (Nano ZS-90, Malvern Panalytical, Worcestershire, UK). Surface chemistry was analyzed by attenuated total reflectance Fourier-transform infrared spectroscopy over 7800–350 cm⁻^1^ (Nicolet iS10, Thermo Fisher Scientific).

### Animal grouping, design and treatment

Male C57BL/6 mice (6–8 weeks) were obtained from the Experimental Animal Center of Xiamen University and maintained under a 12 h light–dark cycle (22 ± 2 °C, 45–65% humidity) with *ad libitum* access to standard chow. Following a 7-day acclimation period, mice were randomly allocated to six groups (*n* = 5 cages, one mouse per cage to avoid coprophagy-driven microbiota transfer): control (Ctrl, 200 µL sterile saline, daily gavage); PBAT-MPs low-dose (PBAT-MPs-L, 0.5 mg kg^−1^, daily gavage for 28 days); PBAT-MPs medium-dose (PBAT-MPs-M, 5 mg kg^−1^, daily gavage for 28 days); PBAT-MPs high-dose (PBAT-MPs-H, 50 mg kg^−1^, daily gavage for 28 days); PBAT-MPs + FMT (PBAT-MPs as above plus fecal microbiota transplantation, 200 µL fresh suspension, gavage every 48 h on days 15–28); and PBAT-MPs + butyrate (PBAT-MPs as above plus 200 mM sodium butyrate in drinking water, refreshed every 48 h). The 200 mM sodium butyrate concentration was selected based on prior mouse studies showing good oral tolerability. To control for potential confounding from sodium load and solution acidity, control drinking water was sodium-matched with NaCl and pH-adjusted (and verified) to match the sodium butyrate solution. Body weight and fluid intake were recorded daily. All procedures were approved by the Animal Experiment Ethics Committee of Xiamen University (approval No. XMULAC20200201) and were conducted in accordance with ARRIVE 2.0 guidelines.

### Fecal microbiota transplantation (FMT)

Fresh fecal pellets were collected from control mice within 30 min, pooled, and homogenized (1:10 w/v) in sterile saline. The suspension was sequentially filtered through sterile 200-, 400-, and 800-mesh sieves to remove debris, then centrifuged at 1000 × g, 4 °C, for 5 min. The resulting bacterial pellet was re-suspended in saline containing 10% (v/v) sterile glycerol, aliquoted (1 mL), snap-frozen in liquid nitrogen, and stored at −80 °C for up to 8 weeks [24]. Before transplantation, aliquots were thawed on ice and administered by oral gavage (200 µL per mouse).

### Behavioral phenotyping

All behavioral testing was performed between 09:00 and 14:00 under ~30 lx red light. Mice were acclimated to the testing room for at least 1 h, and all apparatuses were cleaned with 75% ethanol between trials. For open-field (OF) test: Each mouse was placed in the center of a square arena (42 cm L × 42 cm W × 40 cm H) and allowed to explore freely for 5 min. Total distance travelled was recorded using TopScanLite (Clever Sys Inc., Reston, VA, USA). For Y-maze test: Mice were allowed to explore the maze freely for 5 min. Arm entries and spontaneous alternations were recorded, and alternation percentage was calculated accordingly. For novel-object recognition (NOR) test: Time spent exploring novel and familiar objects was recorded [25]. Recognition index (RI) was calculated as RI = T_novel_ / (T_novel_ + T_familiar_), where T_novel_ and T_familiar_ denote exploration time (s) for novel and familiar objects, respectively.

### Fecal water content

Mice were individually housed in clean, ventilated plastic cages, and all fecal pellets excreted within 2 h were collected immediately and weighed to 0.1 mg precision (wet weight, Ww). Pellets were dried to constant mass at 65 °C for 24 h and re-weighed (dry weight, Wd). Fecal water content (%) was calculated as [(Ww – Wd)/Ww] ⋅ 100 [24].

### Ex vivo fluorescence imaging of organs

Immediately after euthanasia, brain, colon, liver, kidney, and lung were harvested, rinsed in ice-cold PBS, and gently blotted dry. PBAT-MPs fluorescence (λex = 630 nm, λem = 680 nm) was imaged using an IVIS Spectrum system (PerkinElmer, Waltham, MA, USA). Imaging parameters, including exposure time and binning, were kept constant across all samples. Tissue autofluorescence was corrected by subtracting signal obtained from organ-matched unexposed controls [26].

### Histopathology

Serial 4-µm paraffin sections of brain and colon were cut and mounted on adhesive slides. For general morphology, sections were stained with hematoxylin and eosin (H&E, Beyotime) following manufacturer’s instructions. Neuronal integrity was evaluated on adjacent brain sections stained with Nissl stain (Beyotime), and goblet-cell density was assessed on colon sections stained with periodic acid–Schiff (PAS, Beyotime). All bright-field images were acquired using an inverted microscope (Nikon, Tokyo, Japan) under identical illumination/exposure settings across groups, and all quantifications were performed in a blinded manner using Fiji/ImageJ.

For each sample, five randomly selected, non-overlapping fields of view (FOVs) were acquired. Neuronal counting was performed using the Cell Counter plugin in ImageJ. Neurons were identified by standard morphological criteria (large round nucleus, loose chromatin, clear nucleolus, abundant cytoplasm, and visible basophilic Nissl bodies). Area (mm²) of each FOV was calculated based on scale bar, and neuronal density was reported as cells/mm² (total counted neurons divided by total analyzed area). Colonic H&E staining was scored as follows: 0, no mucosal damage; 1, discrete epithelial injury; 1.5, superficial mucosal erosion; and 2, extensive mucosal damage extending into deep intestinal structures. Goblet cells were identified by typical PAS-positive morphology (cup-shaped/oval epithelial cells with magenta cytoplasm and basally located nuclei) and quantified as goblet-cell number per unit area using ImageJ.

### Immunofluorescence (IF) staining

BODIPY™ 493/503 was applied to cells or brain cryosections at recommended dilution and incubated at 37 °C for 30 min in the dark. Nuclei were counterstained with Hoechst 33342 [27]. For paraffin-embedded brain tissue sections, antigen retrieval was performed using sodium citrate buffer (Beyotime). Sections were permeabilized with 0.3% (v/v) Triton X-100 (Sigma-Aldrich) for 5 min, blocked with 1% (w/v) bovine serum albumin (BSA; Sigma-Aldrich) for 2 h, and incubated sequentially with primary and secondary antibodies (listed in Supplementary Table S1). Nuclei were counterstained with DAPI. Fluorescence images were acquired on a laser scanning confocal microscope (LSM 780; Zeiss, Oberkochen, Germany) using identical settings across groups, and quantification was performed in a blinded manner using Fiji/ImageJ (and Imaris where indicated).

Mean fluorescence intensity (MFI) of Iba1, GFAP, SOX10, and ATF4 was quantified in ImageJ. Channels were split and converted to 8-bit grayscale images. ROIs were defined based on DAPI nuclear staining and expanded to include cytoplasmic area; MFI was measured within each ROI. For colocalization-related analyses, MFI was measured within each ROI for p-eIF2α versus total eIF2α, and for overlap channels (Iba1^+^Plin2, Iba1^+^eIF2α, and Iba1^+^p-eIF2α) versus Iba1. The following ratios were calculated: p-eIF2α/eIF2α, Iba1^+^Plin2/Iba1, Iba1^+^eIF2α/Iba1, and Iba1^+^p-eIF2α/Iba1. At least three non-consecutive sections per animal were analyzed, and results are presented as mean ± SD. Lipid accumulation in microglia was quantified as LD area within Iba1-positive (Iba1^+^) microglia (reported as LD area per cell, and/or as percentage of LD-positive Iba1^+^ cells where applicable), with identical thresholds and analysis settings applied across groups. Neuronal morphometry was quantified as axon length, dendritic length, and dendrite number.

### Transmission electron microscopy (TEM)

Mouse brain tissues and HMC-3 cells were fixed with 2.5% (v/v) glutaraldehyde and 4% (w/v) paraformaldehyde, post-fixed in 1% (w/v) osmium tetroxide, and embedded in epoxy resin. Ultrathin sections (70–80 nm) were stained with uranyl acetate and lead citrate, and examined using TEM (Tecnai 20; FEI, Hillsboro, OR, USA). Microglia were identified based on established ultrastructural criteria [28], including irregularly shaped nucleus with condensed/peripheral heterochromatin, relatively scant cytoplasm with thin cellular processes, and abundant lysosomes, whilst excluding neuronal-like profiles characterized by large euchromatic nucleus with prominent nucleolus and abundant rough endoplasmic reticulum/Nissl-like structures. Only cells meeting these criteria were included for representative micrographs and quantitative analyses. For each sample, five non-overlapping microglia-containing FOVs were randomly captured, and 3–5 microglial cell profiles were analyzed per FOV (~15–25 microglial profiles per sample). Ultrastructural lipid accumulation was quantified in a blinded manner using ImageJ as total LD area per FOV (and reported with corresponding unit).

### Isolation of mouse primary microglia

Primary microglia were isolated as described previously [15]. Briefly, mice were transcardially perfused with Medium A (HBSS supplemented with 15 mM HEPES, 0.05% (w/v) glucose, and DNase I at 1:500 dilution). Hippocampi were dissected and dissociated into single-cell suspensions. Cells were stained with CD11b-PE and CD45-APC antibodies at room temperature for 30 min, centrifuged at 400 × g for 5 min, and resuspended in PBS. LDs were labeled with BODIPY™ 493/503 at 37 °C for 30 min in the dark. BODIPY^high^ microglia were sorted using a MoFlo Astrios EQ flow cytometer (Beckman Coulter, Brea, CA, USA).

### Primary neuron isolation from embryonic mice

Timed-pregnant mice at embryonic days 16–18 (E16–18) were euthanized, and embryos were aseptically removed. Fetal brains were dissected after decapitation, and meninges and blood vessels were carefully removed under a stereomicroscope. Hippocampi were isolated and digested with papain (2 mg mL⁻¹) at 37 °C for 30 min. Tissues were gently triturated to obtain a single-cell suspension, filtered through a 40 μm cell strainer, and centrifuged at 300 × g for 5 min. Cells were resuspended in neuron-specific culture medium, counted, and plated onto poly-L-lysine-coated culture vessels. Cultures were maintained at 37 °C in a humidified incubator with 5% CO_2_. Quantitative analysis of TUJ1 staining in primary neurons was performed under identical confocal settings. For each sample, five non-overlapping FOVs were randomly captured, and 5–10 neurons were analyzed per field (~25–50 neurons per sample per condition). Neurite morphology was analyzed using the NeuronJ plugin in ImageJ, including axon length per neuron, total dendritic length, and dendritic branch count.

### 16S rRNA sequencing and analysis of mouse gut microbiome

Fecal samples were collected immediately after euthanasia, snap-frozen in liquid nitrogen, and stored at −80 °C until analysis. Microbial genomic DNA was extracted, quality-checked, and used for amplification of V3–V4 region of bacterial 16S rRNA gene. Purified amplicons were used for library preparation and sequenced on an Illumina NovaSeq 6000 platform (Illumina, San Diego, CA, USA) at APT Biotechnology Co., Ltd. (Shanghai, China). Raw reads were quality-filtered, denoised, and resolved into amplicon sequence variants (ASVs). Alpha- and beta-diversity, taxonomic profiling, LEfSe (linear discriminant analysis effect size), and functional prediction were performed using QIIME2 and R packages [29].

### Untargeted metabolomics of mouse feces

Untargeted metabolomics analysis was performed by APT Biotechnology Co., Ltd. Fecal samples were homogenized in methanol**–**water (1:4, v/v) containing L-2-chlorophenylalanine (0.3 mg mL⁻¹) as an internal standard using ultrasonic disruption. After centrifugation, the supernatant was collected, dried, and reconstituted in methanol**–**water (1:4, v/v) for LC-MS analysis. LC-MS data were processed using Progenesis QI (v2.3). Metabolites were identified against HMDB and METLIN databases. Differentially expressed metabolites (DEMs) were identified by orthogonal partial least-squares discriminant analysis (OPLS-DA) with variable importance in projection (VIP) score > 1, followed by KEGG-based pathway annotation.

### Western blotting (WB)

Proteins were extracted by SDS-heat lysis, separated by SDS-PAGE, and transferred onto PVDF membranes. After blocking with 5% (w/v) BSA for 1 h at room temperature, membranes were incubated with primary and secondary antibodies (listed in Supplementary Table S2), and visualized using an Azure C300 system (Azure Biosystems, Beijing, China).

### Quantitative real-time polymerase chain reaction (qRT-PCR)

Total RNA was extracted with TRIzol reagent (Ambion, Austin, TX, USA) and reverse-transcribed using PrimeScript™ RT kit (TaKaRa, Shiga, Japan). qRT-PCR was performed using primers listed in Supplementary Table S3.

### Plasmid construction and cell transfection

HMC-3 cells were transduced with pBabe-Rptor lentivirus and selected to establish Rptor-overexpressing (Rptor-OE) stable lines.

### Flow cytometry (FCM)

After treatment, cells were incubated with BODIPY™ 493/503 at 37 °C for 30 min in the dark, washed with PBS, and collected for analysis on a CytoFLEX flow cytometer (Beckman Coulter).

### Enzyme-linked immunosorbent assay (ELISA)

Levels of IL-6, IL-1β, TNF-α, LPS, ceramide, and butyrate were measured using commercial ELISA kits according to the manufacturers’ instructions. Absorbance was read on a multifunctional microplate reader (BMG LABTECH, Offenburg, Germany), and concentrations were calculated from standard curves.

### Statistical analysis

Data are presented as the mean ± standard deviation (SD). Statistical analyses were performed using Student’s *t*-test or one-way analysis of variance (ANOVA) followed by Tukey’s post hoc test, as appropriate. Correlations were assessed using Spearman’s rank correlation analysis. A *P*-value < 0.05 was considered statistically significant. Analyses and graphs were generated using GraphPad Prism 7.0 (GraphPad Software, San Diego, CA, USA). Figures were assembled using CorelDRAW 2024 (Corel Corporation, Ottawa, Canada).

## Results

### FMT attenuates PBAT-MPs-induced colonic inflammation and hippocampal neurodegeneration in mice

To probe microbe–particle interactions, we first employed an in vitro simulated intestinal digestion model to assess how gut microbiota affect PBAT-MPs. As shown in Fig. S1A–E, microbial digestion converted spherical PBAT-MPs into smaller fragments, reducing hydrodynamic diameter from 1.3 μm to 320 nm (Fig. S1A–B). Approximately 34% of PBAT-MPs were biodegraded over 4 weeks (Fig. S1C). Zeta potential increased from −27 mV to −11 mV (Fig. S1D), indicating significantly reduced colloidal stability. FTIR spectra showed markedly attenuated C–H and –C=O stretching vibrations (Fig. S1E), consistent with disruption of the PBAT-MPs chemical structure. Together, these data indicate that gut microbiota actively participate in PBAT-MPs biodegradation, supporting robust microbe–particle interactions.

**Fig. 1:**
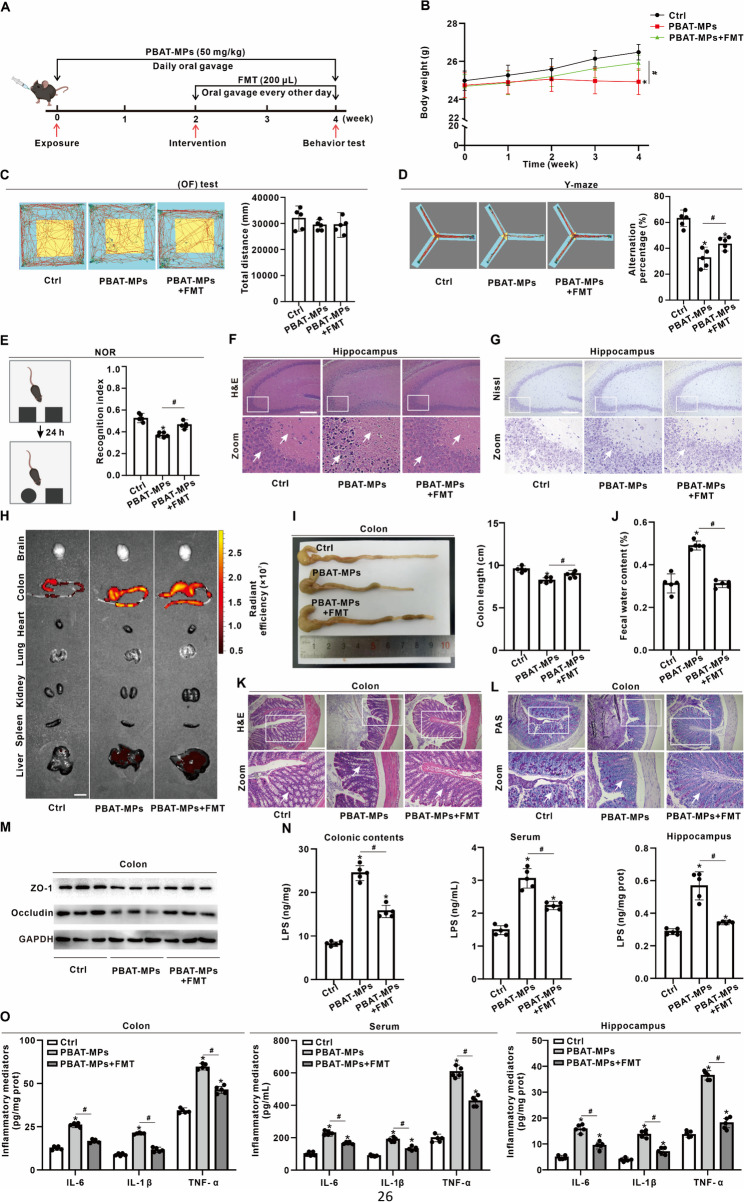
FMT attenuates PBAT-MPs-induced colonic inflammation and hippocampal neurodegeneration in mice. C57BL/6 mice received daily oral gavage of PBAT-MPs (50 mg kg⁻¹) for 4 weeks, followed by treatment with or without FMT for 2 weeks. *n* = 5 per group. **A **Flow chart of animal experiment design. **B **Body weight during treatment; *n* = 5. **C **Representative track images in open-field test (left); total distance (right); *n* = 5. **D **Representative track images in Y-maze test (left); spontaneous alternation percentage (right); *n* = 5. **E **Schematic diagram of novel-object recognition test (left); recognition index (right); *n* = 5. **F **Representative H&E-stained images of hippocampal CA3; scale bar, 100 μm. **G **Representative Nissl-stained images of hippocampal CA3; scale bar, 100 μm. **H **Representative ex vivo fluorescence images showing PBAT-MPs accumulation in major organs; scale bar, 1 cm. **I **Representative images of colon tissue (left); colon length analysis (right); *n* = 5. **J **Water percentage of fecal pellets;  *n* = 5. **K **Representative H&E-stained images of colon tissue; scale bar, 100 μm. **L **Representative PAS-stained images of colon tissue; scale bar, 100 μm. **M** Protein levels of ZO-1 and Occludin detected by WB; *n* = 3. **N **LPS levels in colonic contents, serum, and hippocampal tissue measured by ELISA; *n* = 5. **O **IL-6, IL-1β, and TNF-α levels in colon tissue, serum, and hippocampal tissue measured by ELISA; *n* = 5. * *P* < 0.05 versus control; # *P* < 0.05 versus corresponding group

To evaluate PBAT-MPs neurotoxicity and dissect underlying gut–brain axis mechanisms, C57BL/6 mice received a 4-week oral gavage regimen; fecal microbiota transplantation (FMT) was subsequently performed to verify gut microbiome contribution (Fig. 1A). In addition to the original high-dose exposure paradigm, we conducted a 4-week dose–response experiment using low, medium, and high PBAT-MPs doses (0.5, 5, and 50 mg kg⁻¹ day⁻¹) to better define the exposure threshold (Fig. S2A). This analysis revealed a graded, dose-dependent pattern, with high-dose group showing a modest but significant body weight reduction by week 4 (Fig. S2B). Behavioral outcomes exhibited differential sensitivity: Y-maze spontaneous alternation decreased at medium dose and was more pronounced at high dose, whereas reduced open-field locomotor activity and impaired novel object recognition were evident mainly at high dose (Fig. S2C–E). Consistently, overt hippocampal injury (H&E/Nissl; reduced neuronal density) and colitis-like phenotypes (shortened colon length, elevated fecal water content and histological score) were predominantly observed in high-dose group, whilst low- and medium-dose groups showed minimal or no significant histopathological changes under present conditions (Fig. S2F–K).

In main in vivo expneriments, 4 week PBAT-MP exposure significantly reduced body weight (−12.2% vs. control), an effect largely reversed by FMT (Fig. 1B). In open-field test, total distance did not differ significantly among groups, indicating no clear locomotor activity change (Fig. 1C). By contrast, cognitive performance was markedly impaired: Y-maze spontaneous alternation rate decreased by 38% in PBAT-MP-exposed mice (Fig. 1D), and novel object recognition index decreased by 31% (Fig. 1E), indicating deficits in spatial working memory and recognition memory, respectively. Both impairments were substantially ameliorated by FMT. Given hippocampal centrality in memory processing, histopathological analyses focused on this region. H&E staining revealed prominent alterations, including pyknotic nuclei, in the CA3 subfield of PBAT-MP-exposed mice (Fig. 1F; Fig. S3A), whilst Nissl staining showed marked reduction in Nissl substance and neuronal density (Fig. 1G; Fig. S3B). These pathological changes were significantly attenuated by FMT, supporting a critical role for gut microbiota homeostasis in modulating PBAT-MP-induced neurodegenerative injury.

Ex vivo imaging demonstrated that orally administered PBAT-MPs predominantly accumulated in colon, with lower levels in liver, whereas no fluorescent signal was detected in brain or other peripheral organs (Fig. 1H). This distribution pattern supports the view that PBAT-MPs exert neurotoxicity indirectly via gut–brain axis signaling, rather than through direct CNS entry. Consistent with intestinal injury, PBAT-MP exposure significantly shortened colon length (Fig. 1I) and increased fecal water content (Fig. 1J), both indicative of colitis-like changes. Histological analysis further revealed crypt atrophy, goblet cell depletion, and reduced mucin secretion (Fig. 1K–L; Fig. S3C–D). In parallel, tight-junction proteins ZO-1 and Occludin were markedly downregulated (Fig. 1M), indicating impaired epithelial barrier integrity. Because luminal LPS and inflammatory mediators are key drivers of gut and systemic inflammation and major effectors of the gut–brain axis [30], we measured LPS, IL-6, IL-1β, and TNF-α in colon, serum, and hippocampus. Compared with controls, PBAT-MP exposure significantly increased all these inflammatory markers at each site (Fig. 1N–O). Notably, FMT markedly attenuated these changes, supporting a microbiota-dependent mechanism underlying both intestinal and hippocampal injury. Collectively, these findings indicate that, following oral exposure, PBAT-MPs are retained primarily in colon, where they disrupt epithelial homeostasis and provoke local and systemic inflammation, ultimately contributing to hippocampal injury through the gut–brain inflammatory axis.

### PBAT-MPs exposure drives microglial lipotoxicity via gut–brain axis signaling originating from colonic inflammation

**Fig. 2:**
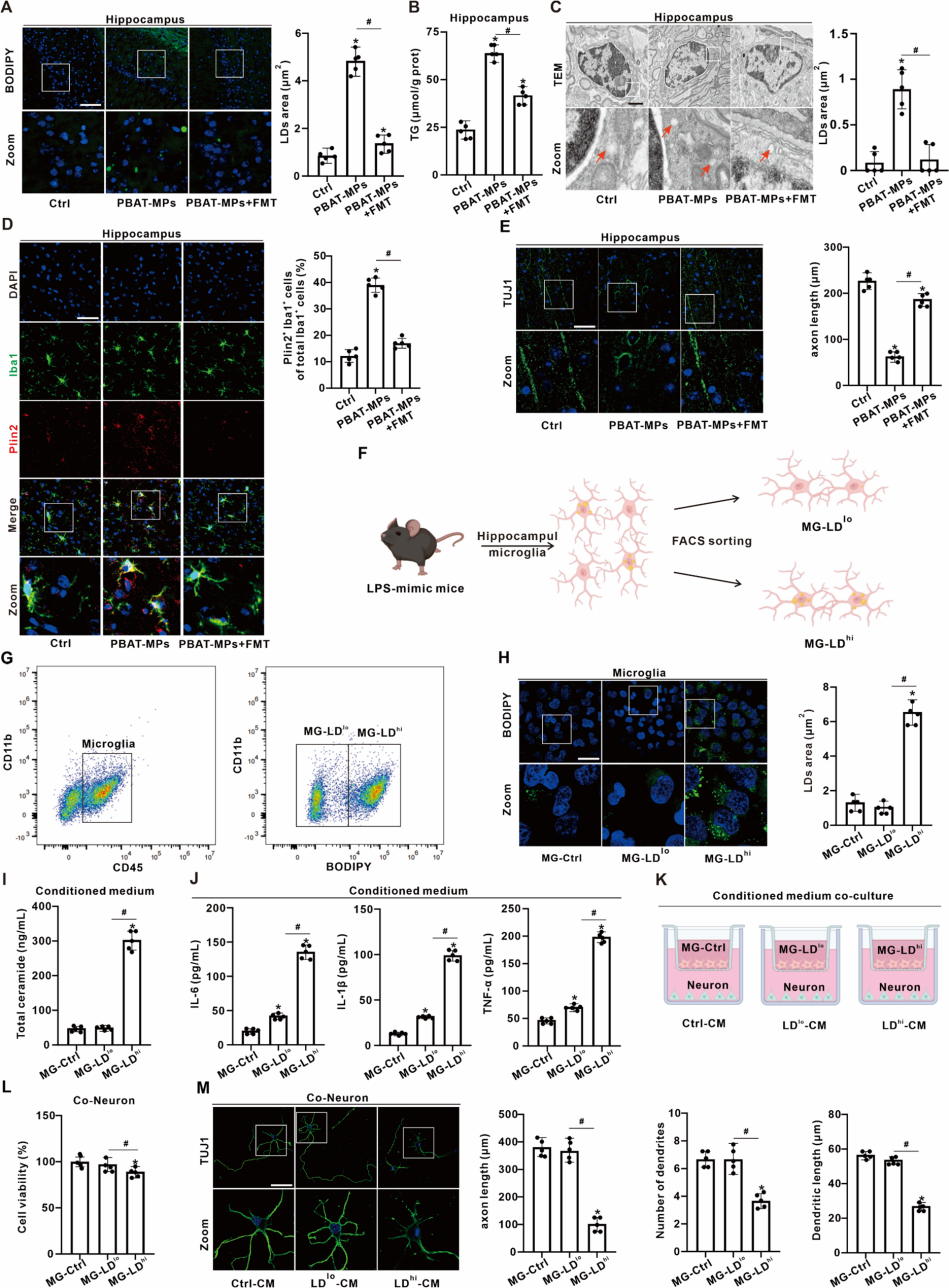
PBAT-MPs exposure drives microglial lipotoxicity via gut–brain axis signaling originating from colonic inflammation. **A–E** C57BL/6 mice received daily oral gavage of PBAT-MPs (50 mg kg⁻¹) for 4 weeks, followed by treatment with or without FMT for 2 weeks. *n* = 5 per group. **A **Representative confocal images of BODIPY™ 493/503 staining in hippocampal CA3 (left); scale bar, 50 μm. Quantification of LDs area (right). **B **Triglyceride (TG) levels in hippocampal tissue. **C **Representative TEM images of LDs structures in microglia; scale bar, 5 μm. **D **Representative IF images of Plin2 (red) and Iba1 (green) in hippocampal CA3 (left); scale bar, 50 μm. Quantification of Plin2^+^ and Iba1^+^ cells (right). **E **Representative IF images of TUJ1 (green) in hippocampal CA3 (left); scale bar, 50 μm. Quantification of axon length (right). **F–J **Isolation of BODIPY^low^ (LD-lo) and BODIPY^high^ (LD-hi) microglia from hippocampus of LPS-treated mice (i.p. 1 mg kg⁻¹, 3 days). **F **Flow cytometry sorting strategy. **G **Flow cytometry sorting results. **H **Representative confocal images of BODIPY™ 493/503 staining in microglia (left); scale bar, 10 μm. Quantification of LDs area (right). **I **Ceramide levels in microglial conditioned medium determined by ELISA. **J **IL-6, IL-1β, and TNF-α levels in microglial conditioned medium determined by ELISA. **K–M** Primary neurons co-cultured with LD-lo or LD-hi microglia in a Transwell system for 72 h. **K **Schematic of co-culture paradigms: Ctrl-CM, untreated microglia + neurons; LD^lo^-CM, LD-lo microglia+ neurons; LD^hi^-CM, LD-hi microglia + neurons. **L** Neuronal viability measured by CCK-8 assay. **M **Representative IF images of TUJ1 (green) in co-cultured neurons (left); scale bar, 50 μm. Quantification of axon length, dendrite number, and dendrite length (right). * *P* < 0.05 versus control; # *P* < 0.05 versus corresponding group

Consistent with inflammatory changes observed in Fig. 1, PBAT-MPs induced a pronounced glial response in the hippocampus. Immunofluorescence revealed dose-dependent increases in Iba1 and GFAP, with significant elevations in medium- and high-dose groups (Fig. S4A–B). Multiplex staining further revealed increased GFAP, Iba1, and SOX10 signals following PBAT-MPs exposure, indicating broad glial activation (Fig. S4C). Notably, FMT partially reversed GFAP and Iba1 increases, but not SOX10; independent GFAP staining confirmed partial attenuation of astrocyte reactivity by FMT (Fig. S4D). These findings align with our earlier observation that LPS, IL-6, IL-1β, and TNF-α levels were significantly elevated in colon, serum, and hippocampus following PBAT-MPs exposure and markedly reduced by FMT (Fig. 1N–O), supporting a gut microbiota-dependent inflammatory mechanism underlying intestinal and hippocampal injury. Although astrocytes were also affected, we focused on microglia because they are central effectors of neuroinflammation and showed a clearer microbiota-dependent response. PBAT-MPs caused a 5.1-fold increase in BODIPY™ 493/503-positive lipid droplet area in hippocampal CA3 (Fig. 2A) and significantly elevated hippocampal triglyceride content (Fig. 2B). TEM and PLIN2/Iba1 co-staining further showed that lipid droplets preferentially accumulated in microglia (Fig. 2C–D). Functionally, PBAT-MPs reduced cumulative axonal length by 72% (Fig. 2E), whereas FMT largely reversed both microglial lipid accumulation and axonal injury, highlighting a key role for gut microbiota in PBAT-MPs-induced microglial lipotoxicity and secondary neuronal damage.

To examine the contribution of gut-derived inflammation, we administered low-dose LPS (1 mg kg^−1^, i.p.) to mimic PBAT-MPs-induced intestinal inflammation. Hippocampal microglia were subsequently isolated and sorted by flow cytometry into BODIPY^low^ (LD-lo) and BODIPY^high^ (LD-hi) populations (Fig. 2F–G). Following sorting, LD-hi microglia showed a 6.5-fold increase in intracellular LD area (Fig. 2H). ELISA further showed that conditioned medium from LD-hi microglia contained significantly higher levels of total ceramide (Fig. 2I) and pro-inflammatory cytokines IL-6, IL-1β, and TNF-α (Fig. 2J). To assess their neurotoxic potential, LD-lo and LD-hi microglia were co-cultured with neurons in a Transwell system (Fig. 2K). Compared with LD-lo cells, LD-hi microglia reduced neuronal viability by 12.5% (Fig. 2L) and induced marked neuritic damage, including 72% reduction in primary axon length, 43% decrease in dendrite number, and 52% reduction in total dendritic length (Fig. 2M). Collectively, these findings indicate that PBAT-MPs instigate intestinal inflammation, thereby propagating gut–brain axis that reprogrammes microglial lipid metabolism. The resulting lipotoxic and pro-inflammatory microglial phenotype promotes neuronal injury, providing a mechanistic link between BMP exposure and neurodegenerative pathology.

### Gut microbiota-derived butyrate inversely correlates with microglial lipotoxicity

**Fig. 3:**
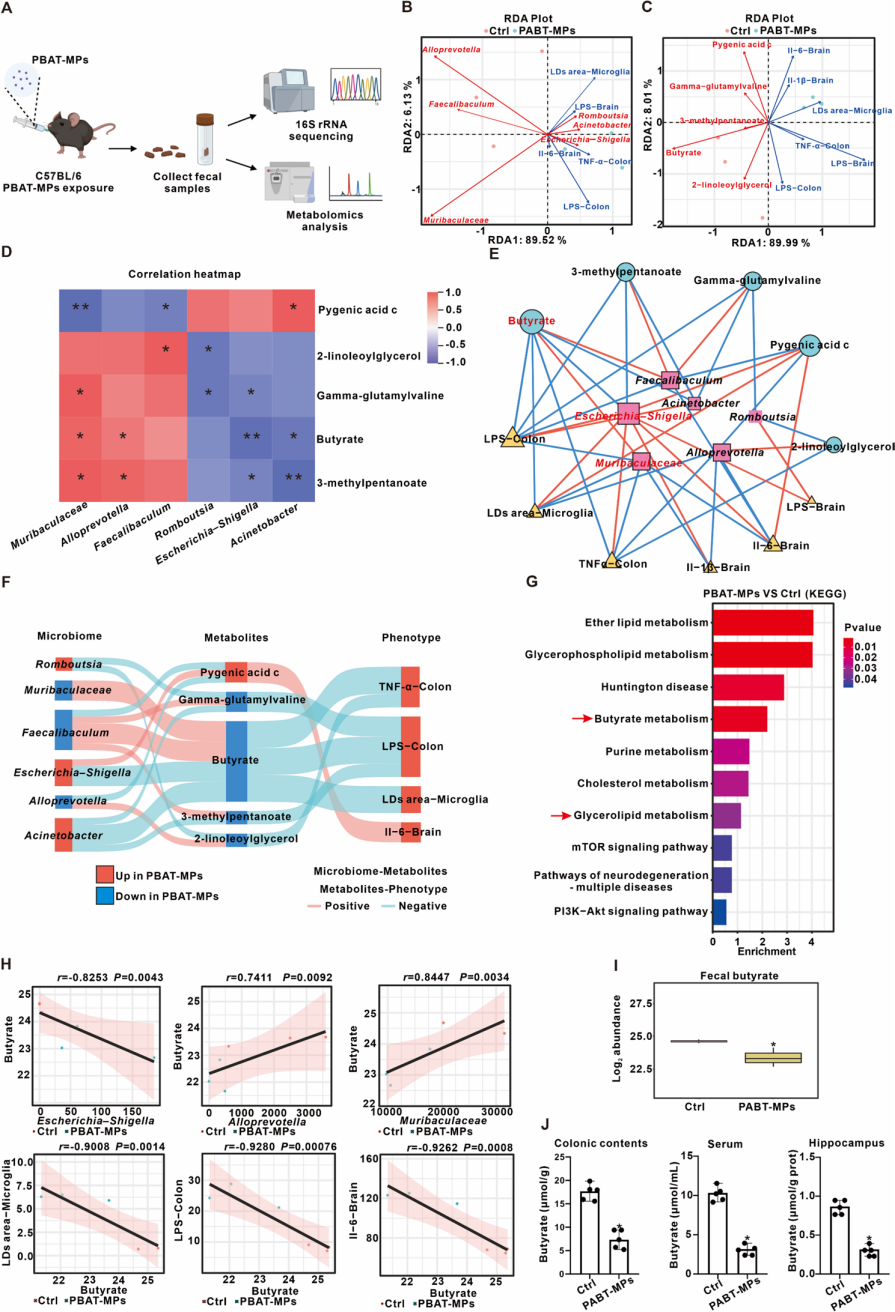
Gut microbiota-derived butyrate inversely correlates with microglial lipotoxicity. Fecal samples from PBAT-MPs-exposed and control mice (*n* = 3) were collected for 16S rRNA sequencing and untargeted metabolomics. **A **Schematic of fecal multi-omics workflow. **B **RDA showing linear correlations between differential gut microbiota and phenotypes; acute angle = positive, obtuse = negative, right = no correlation; arrow length reflects effect strength. **C **RDA showing linear correlations between differential gut metabolites and phenotypes. **D **Correlation heat map of differential microbes and metabolites. **E **Network analysis among phenotypic parameters, differential microbiota, and metabolites; red lines = positive, blue lines = negative correlations. **F **Sankey diagram linking phenotypic parameters to differential microbiota and metabolites. **G **KEGG-based enrichment of differential metabolic pathways. **H** Correlation analysis of butyrate with *Escherichia*–*Shigella*,* Alloprevotella*,* Muribaculaceae*, LDs area-microglia, LPS-colon, and IL-6-brain; linear regression R and *P* values shown. **I **Inter-group differences in butyrate. **J **Butyrate levels in colonic contents, serum, and hippocampal tissue measured by ELISA. * *P* < 0.05 versus control

To define how gut microbiota drive microglial lipid overload along the gut–brain axis, we integrated 16S rRNA sequencing with untargeted fecal metabolomics (Fig. 3A). PBAT-MP exposure did not significantly alter α-diversity, as ACE, Chao1, and Shannon indices remained unchanged (Fig. S5A–C). In contrast, β-diversity analysis based on Bray–Curtis dissimilarity showed clear separation between PBAT-MP-exposed and control mice (PERMANOVA: R² = 0.286, *P* < 0.05; Fig. S5D). At genus level, PBAT-MPs significantly reduced the anti-inflammatory fibre-degraders *Muribaculaceae* and *Alloprevotella* whilst enriching pro-inflammatory taxa *Escherichia*–*Shigella* and *Acinetobacter* (Fig. S5E–H). KEGG functional prediction of differentially abundant taxa indicated enrichment of pathways related to LPS biosynthesis, pathogenic *Escherichia coli* infection, and shigellosis, together with suppression of butyrate metabolism and enhancement of glycerolipid metabolism (Fig. S5I). These findings suggest a microbial shift toward a pro-inflammatory state associated with impaired barrier function and disturbed host lipid metabolism. To identify microbiota-dependent metabolites that may mediate these effects, we performed untargeted LC–MS/MS metabolomics. OPLS-DA achieved robust separation between groups (Q² = 0.761; Fig. S5J). Using VIP > 1.0 and *P* < 0.05 as selection criteria, we identified 11 discriminatory metabolites (Fig. S5K–L). Notably, fecal butyrate was down-regulated in PBAT-MPs-exposed mice, mirroring predicted suppression of microbial butyrate metabolism. These data position butyrate loss as a candidate metabolic cue linking dysbiosis to microglial lipotoxicity.

Redundancy analysis (RDA) further demonstrated significant associations among differential bacterial genera, their metabolites, inflammatory indices, and microglial LD burden (Fig. 3B–D). Integration of these variables generated a microbe–metabolite–phenotype interaction network (Fig. 3E–F), in which *Escherichia*–*Shigella* and *Acinetobacter* were negatively correlated with butyrate, whereas *Muribaculaceae* and *Alloprevotella* showed positive correlations. Butyrate, in turn, was negatively associated with colonic TNF-α, LPS, and hippocampal microglial LD deposition area. KEGG enrichment of differential metabolites likewise showed down-regulation of butyrate metabolism and up-regulation of glycerolipid metabolism (Fig. 3G). Linear regression confirmed robust dose–response relationships: butyrate plotted against *Escherichia*–*Shigella*, *Alloprevotella*, *Muribaculaceae*, microglial LD area, colonic LPS, and brain IL-6 yielded R² ≥ 0.549 and *P* ≤ 0.01 for every predictor (Fig. 3H). LC–MS/MS metabolomics confirmed significant decrease in fecal butyrate (Fig. 3I), and butyrate levels in colon, serum, and hippocampus mirrored this decline (Fig. 3J). Collectively, expansion of LPS-producing pathogens coupled with depletion of butyrate-producing commensals lowers systemic butyrate availability, thereby removing a brake on microglial lipid accumulation and neuroinflammation. These data establish butyrate as a quantitative transducer of gut-derived signals that restrain PBAT-MPs-induced microglial lipotoxicity.

### Butyrate alleviates microglial lipotoxic injury by suppressing ISR-mediated lipid droplet biogenesis

To investigate the role of butyrate in microglial lipotoxicity, we re-analyzed public transcriptomic dataset GSE261007, derived from mouse primary microglia exposed to enteric LPS. Differential gene expression analysis showed marked up-regulation of genes involved in de novo lipogenesis and inflammatory cytokines (Fig. S6A). Consistently, GO, KEGG, and GSEA analyses indicated activation of lipid metabolic and inflammatory response pathways (Fig. S6B–F). Notably, GSEA also revealed significant enrichment of “regulation of response to stress” signature (Fig. S6G). Given that integrated stress response (ISR) promotes microglial lipotoxicity by enhancing lipid synthesis (Fig. S6H) [21], we further examined ISR status. Hierarchical clustering demonstrated coordinated up-regulation of core ISR signaling transcripts (Fig. S6I), accompanied by elevated transcription of canonical ISR target genes (Fig. S6J), confirming robust ISR engagement in enteric LPS-triggered lipotoxic microglia.

**Fig. 4:**
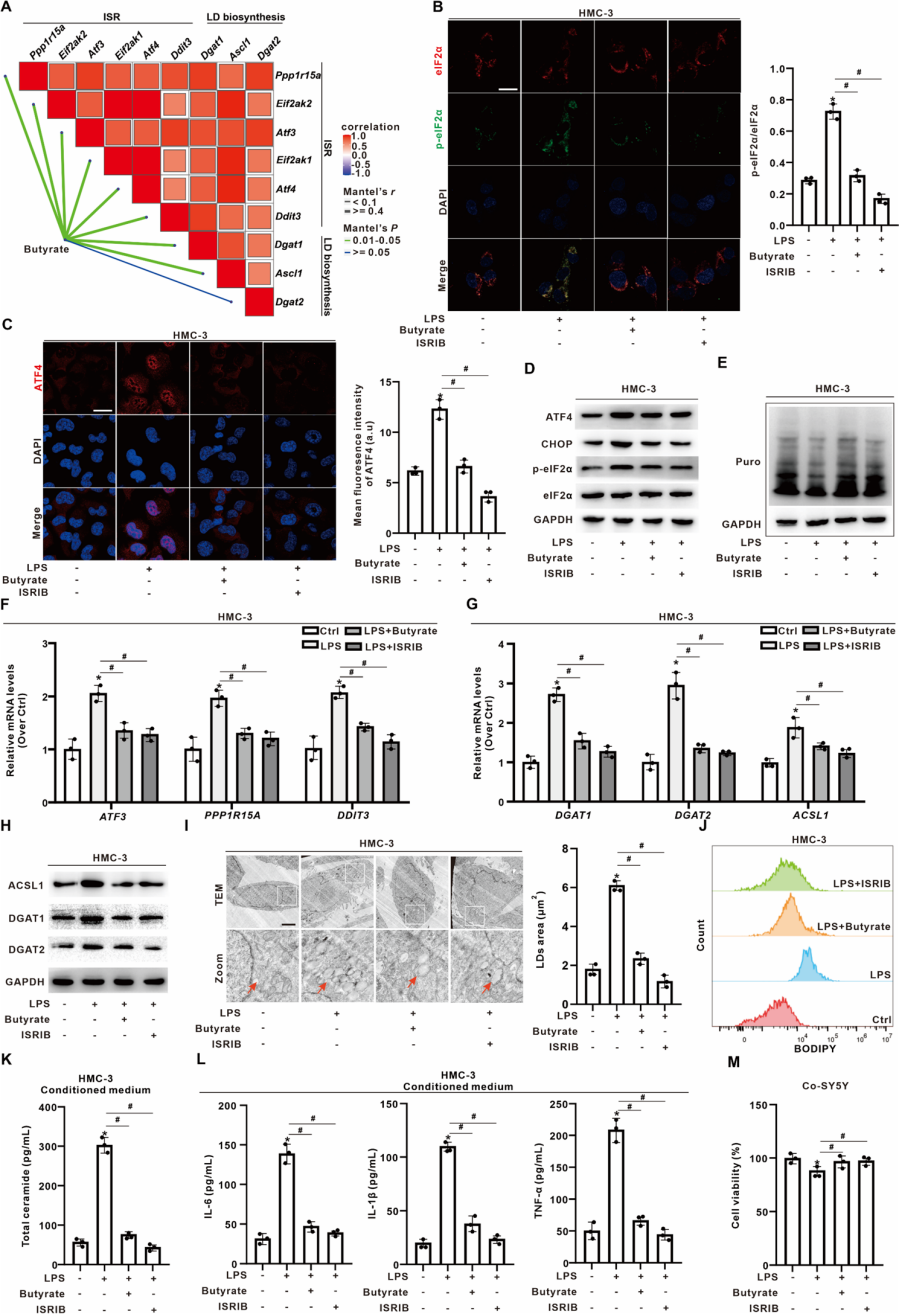
Butyrate alleviates microglial lipotoxic injury by suppressing ISR-mediated lipid droplet biogenesis. **A **Mantel test correlation heat map of butyrate with genes involved in lipid droplet synthesis and ISR-related pathways. **B–L **HMC-3 human microglial cells were exposed to LPS (100 ng mL^−1^) for 72 h and treated with or without butyrate (0.5 mM) or ISRIB (10 nM) for 24 h. **B **Representative IF images of eIF2α (red) and p-eIF2α (green) in HMC-3 (left); scale bar, 10 μm. Quantification of p-eIF2α/eIF2α (right). **C **Representative IF images of ATF4 (red) in HMC-3 (left); scale bar, 10 μm. Quantification of mean fluorescence intensity of ATF4 (right). **D **ISR pathway-related proteins detected by WB. **E** De novo protein synthesis assessed by puromycin (Puro, 1 µM, 30 min) incorporation. **F **mRNA levels of canonical ISR target genes determined by qRT-PCR. **G **mRNA levels of lipid droplet biogenesis genes determined by qRT-PCR. **H **Lipid droplet biogenesis-related proteins detected by WB. **I **Representative TEM images of LD structures in HMC-3 (left); scale bar, 5 μm. Quantification of LD area (right). **J **FCM analysis of BODIPY™ 493/503 staining in HMC-3. **K **Ceramide levels in HMC-3 conditioned medium determined by ELISA. **L** IL-6, IL-1β, and TNF-α levels in HMC-3 conditioned medium determined by ELISA. **M **SH-SY5Y neurons co-cultured in Transwell inserts with HMC-3 cells exposed to LPS (100 ng mL^−1^) for 72 h and treated with or without butyrate (0.5 mM) or ISRIB (10 nM) for 24 h; cell viability measured by CCK-8 assay. * *P* < 0.05 versus control; # *P* < 0.05 versus corresponding group

To explore whether butyrate intersects the ISR–LD synthesis axis, we constructed a Mantel test-based correlation matrix integrating butyrate abundance with transcripts related to LD-biogenesis and ISR signaling. Butyrate levels were negatively correlated with both core ISR genes (e.g., *Atf3*, *Ppp1r15a*, *Ddit3*) and LD-synthetic genes (e.g., *Dgat1*, *Dgat2*, *Acsl1*) (Fig. 4A), prompting the hypothesis that butyrate restrains microglial lipotoxicity via ISR inhibition. We validated this premise in LPS-challenged HMC-3 microglia using selective ISR inhibitor ISRIB as positive control. LPS evoked elevated eIF2α phosphorylation (Fig. 4B), enhanced ATF4 nuclear translocation (Fig. 4C), and up-regulation of core ISR proteins (Fig. 4D). Puromycin incorporation assays revealed marked reduction in nascent protein synthesis (Fig. 4E), whilst qRT-PCR confirmed transcriptional induction of canonical ISR target genes (Fig. 4F). Both butyrate and ISRIB reversed each of these ISR hallmarks.

At lipid metabolic level, LPS significantly increased expression of LD biogenesis enzymes ACSL1 and DGAT1/2 at mRNA and protein levels (Fig. 4G–H); butyrate or ISRIB co-treatment abrogated this induction. TEM showed > 3-fold increase in cytoplasmic LDs following LPS treatment, an effect largely nullified by butyrate or ISRIB intervention (Fig. 4I). Flow cytometry quantification of BODIPY™ 493/503 fluorescence corroborated LD reduction (Fig. 4J). Functionally, butyrate and ISRIB significantly lowered cellular ceramide content (Fig. 4K) and attenuated release of pro-inflammatory cytokines (IL-6, IL-1β, and TNF-α) into microglial conditioned medium (Fig. 4L). In neuron–microglia co-cultures, pre-treatment of microglia with butyrate or ISRIB significantly improved SH-SY5Y neuronal viability under LPS exposure (Fig. 4M). Collectively, these findings indicate that gut-derived inflammatory signals induce microglial lipotoxicity through ISR-dependent transcriptional up-regulation of LD synthetic genes. Butyrate interrupts this pathway by suppressing ISR activation, thereby reducing microglial lipid accretion, neuroinflammatory signaling, and consequent neuronal injury.

### mTORC1 operates upstream of the ISR, and Rptor overexpression blunts butyrate-mediated protection against microglial lipotoxicity

**Fig. 5:**
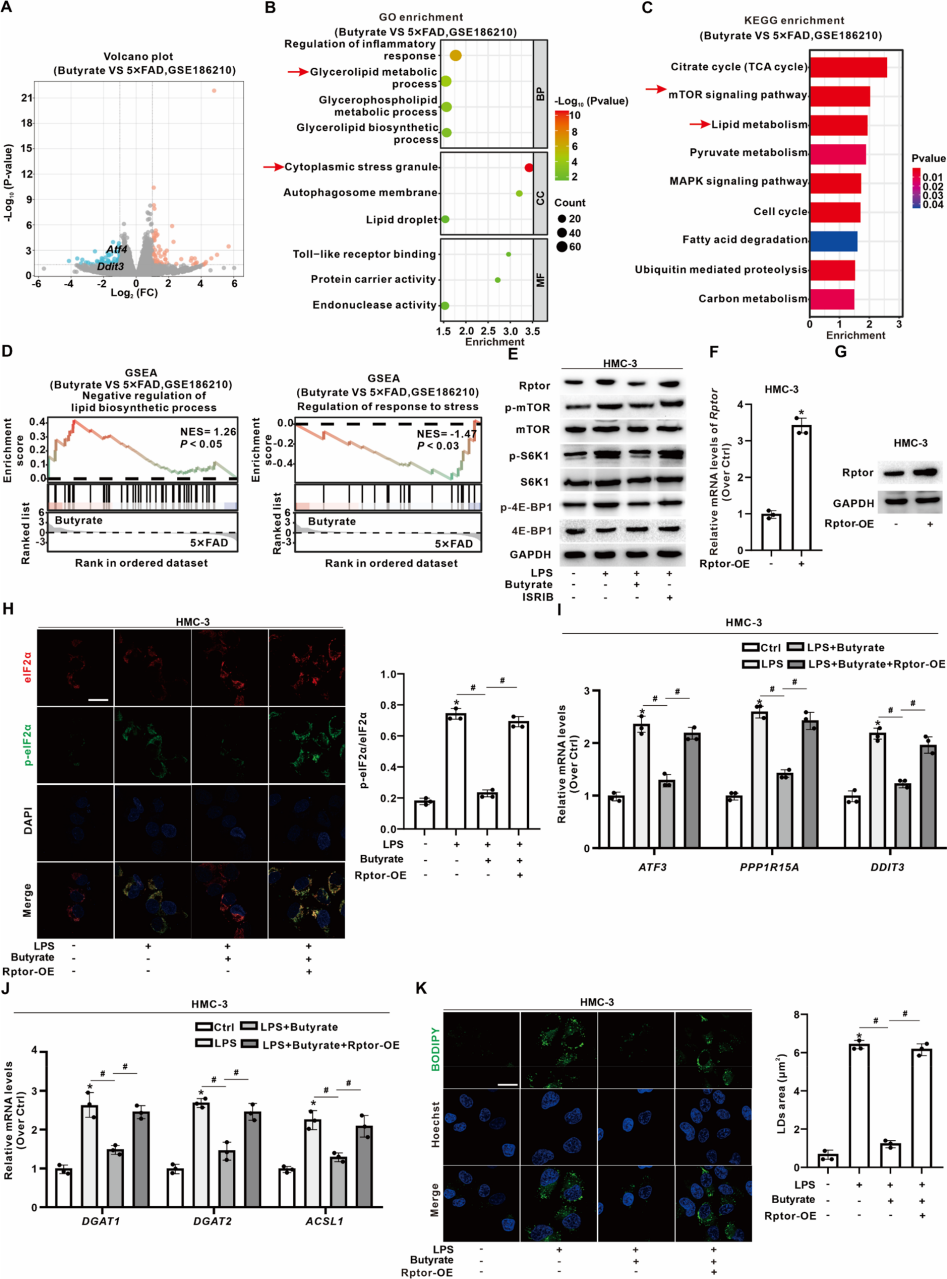
mTORC1 operates upstream of the ISR, and Rptor overexpression blunts butyrate-mediated protection against microglial lipotoxicity. The GSE186210 dataset was derived from RNA-seq data of microglia isolated from butyrate-fed and control 5×FAD mice. *n* = 3 per group. **A **Volcano plot analysis of differentially expressed genes (DEGs); canonical ISR downstream targets highlighted. **B **GO enrichment of DEGs. **C **KEGG enrichment of DEGs. **D **GSEA for gene sets of negative regulation of lipid biosynthetic process and regulation of response to stress. **E **HMC-3 human microglial cells exposed to LPS (100 ng mL^−1^) for 72 h and treated with or without butyrate (0.5 mM) or ISRIB (10 nM) for 24 h; mTORC1 pathway-related proteins detected by WB. **F–K** Stable Rptor-overexpressing (Rptor-OE) HMC-3 cells established and exposed to LPS (100 ng mL^−1^) for 72 h and treated with or without butyrate (0.5 mM) for 24 h. **F** *Rptor* mRNA levels verified by qRT-PCR. **G **Rptor protein levels verified by WB. **H **Representative IF images of eIF2α (red) and p-eIF2α (green) in HMC-3 (left); scale bar, 10 μm. Quantification of p-eIF2α/eIF2α (right). **I **mRNA levels of canonical ISR target genes determined by qRT-PCR. **J **mRNA levels of lipid droplet biogenesis genes determined by qRT-PCR. **K **Representative confocal images of BODIPY™ 493/503 staining in HMC-3 (left); scale bar, 10 μm. Quantification of LD area (right). * *P* < 0.05 versus control; # *P* < 0.05 versus corresponding group

To clarify how butyrate suppresses the ISR, we re-analyzed RNA-seq dataset GSE186210, derived from microglia of 5×FAD mice treated with butyrate. Volcano plot analysis revealed significant down-regulation of canonical ISR target genes (e.g., *Atf4*, *Ddit3*) following butyrate treatment (Fig. 5A). GO, KEGG, and GSEA analyses revealed enrichment of pathways related to negative regulation of lipid biosynthetic process and regulation of response to stress (Fig. 5B–D), consistent with lipid metabolic reprogramming observed in our earlier experiments. Notably, mTOR signaling pathway was also significantly enriched, a pattern further supported by KEGG analysis of differential metabolites (Fig. 5C), implicating mTORC1 as a potential gatekeeper of butyrate action. In LPS-stimulated HMC-3 microglia, Western blotting showed marked mTORC1 activation—as indicated by increased p-mTOR, p-S6K1, and p-4E-BP1—all of which were suppressed by butyrate but not by ISRIB (Fig. 5E). These findings position mTORC1 upstream of ISR signaling in this context.

Previous studies have indicated bidirectional crosstalk between mTORC1 and ISR [31, 32]. To test this hierarchy directly, we established stable Rptor-overexpressing HMC-3 cell lines (3.4-fold elevation at mRNA and protein levels) (Fig. 5F–G). Rptor overexpression markedly attenuated butyrate’s capacity to suppress eIF2α phosphorylation (Fig. 5H), sustained transcription of ISR target genes (Fig. 5I), and preserved expression of LD biogenesis-related enzymes (*DGAT1*, *DGAT2*, *ACSL1*) in the presence of butyrate (Fig. 5J). Confocal imaging of BODIPY™ 493/503 confirmed that Rptor overexpression reversed butyrate-induced suppression of microglial LD formation (Fig. 5K). Together, these results identify mTORC1 as an upstream regulator of the ISR in microglia and demonstrate that enforced mTORC1 activation is sufficient to override the protective effects of butyrate against lipotoxic injury. These findings highlight the mTORC1–ISR axis as a critical pathway through which microbial metabolites regulate microglial lipid metabolism and neuroinflammation.

### Butyrate rescues PBAT-MPs-elicited microglial lipotoxicity and neurodegeneration via the gut–brain axis

**Fig. 6:**
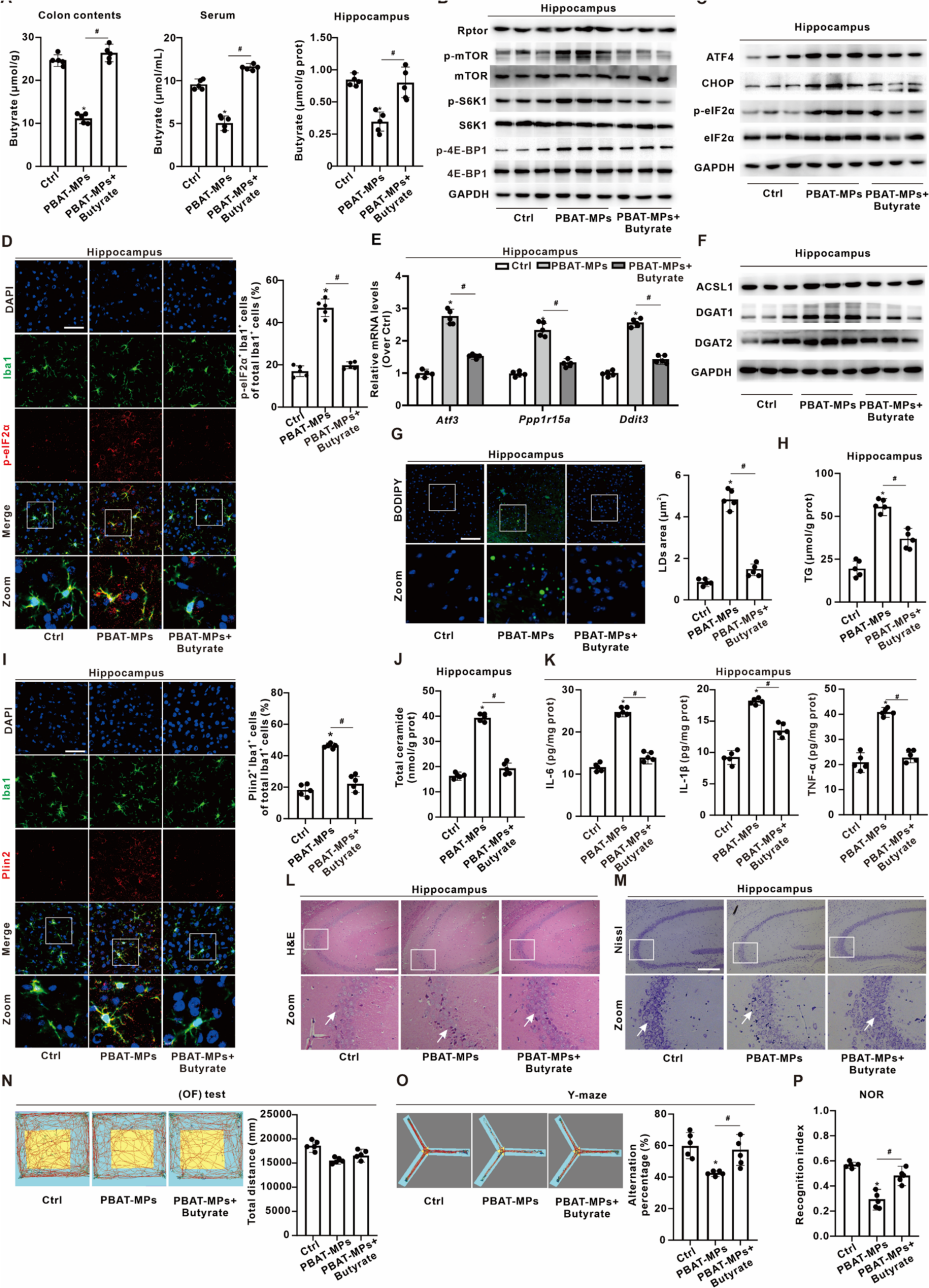
Butyrate rescues PBAT-MPs-elicited microglial lipotoxicity and neurodegeneration via the gut–brain axis. C57BL/6 mice received daily oral gavage of PBAT-MPs (50 mg kg⁻¹) for 4 weeks, followed by intervention with or without butyrate for 2 weeks. *n* = 5 per group. **A **Butyrate levels in colonic contents, serum, and hippocampal tissue measured by ELISA; *n* = 5. **B **mTORC1 pathway-related proteins detected by WB; *n* = 3. **C **ISR pathway-related proteins detected by WB; *n* = 3. **D **Representative IF images of hippocampal sections co-stained for Iba1 (green), p-eIF2α (red), and DAPI (blue) in hippocampal CA3 (left); scale bar, 50 μm. Quantification of p-eIF2α^+^Iba1^+^ cells as percentage of total Iba1^+^ cells (right). **E **mRNA levels of canonical ISR target genes determined by qRT-PCR; *n* = 5. **F **Lipid droplet biogenesis-related proteins detected by WB; *n* = 3. **G **Representative confocal images of BODIPY™ 493/503 staining in hippocampal CA3 (left); scale bar, 50 μm. Quantification of LD area (right). **H **TG levels in hippocampal tissue; *n* = 5. **I **Representative IF images of Plin2 (red) and Iba1 (green) in hippocampal CA3 (left); scale bar, 50 μm. Quantification of Plin2^+^ and Iba1^+^ cells (right). **J **Ceramide levels determined by ELISA; *n* = 5. **K** IL-6, IL-1β, and TNF-α levels determined by ELISA; *n* = 5. **L **Representative H&E-stained images of hippocampal CA3; scale bar, 100 μm. **M **Representative Nissl-stained images of hippocampal CA3; scale bar, 100 μm. **N **Representative track images in open-field test (left); total distance (right). **O **Representative track images in Y-maze test (left); spontaneous alternation percentage (right). **P **Recognition index in novel-object recognition test. * *P* < 0.05 versus control; # *P* < 0.05 versus corresponding group

To validate our in vitro findings in vivo, C57BL/6 mice were exposed to PBAT-MPs with or without butyrate (200 mM) in drinking water for 4 weeks. ELISA quantification demonstrated that butyrate supplementation elevated butyrate levels in colonic contents, serum, and hippocampus (Fig. 6A), confirming effective systemic and central availability. We first examined the intestinal effects of butyrate. Butyrate supplementation reduced luminal abundance of *Shigella*, *Escherichia coli*, and LPS (Fig. S7A–B), suppressed mucosal cytokine release (Fig. S7C), and partially restored tight junction architecture (Fig. S7D–F), indicating improved intestinal barrier integrity.

We next examined neuroprotective effects. WB analysis of hippocampal lysates showed that PBAT-MPs increased phosphorylation of mTORC1 signaling molecules (p-mTOR, p-S6K1, p-4E-BP1) as well as eIF2α; these changes were largely abolished by butyrate co-treatment (Fig. 6B–C). Immunofluorescence in hippocampal CA3 stratum radiatum further confirmed reduced proportion of p-eIF2α^+^ microglia following butyrate treatment (Fig. 6D). Although total eIF2α^+^ microglia did not change significantly (Fig. S7G), canonical ISR target genes (*Atf3*, *Ppp1r15a*, and *Ddit3*) were markedly down-regulated (Fig. 6E).

At lipid metabolic level, butyrate blocked PBAT-MPs-induced up-regulation of LD biogenesis enzymes (ACSL1, DGAT1, DGAT2) (Fig. 6F). Triple labelling with BODIPY™ 493/503, PLIN2 and Iba1 revealed that butyrate lowered LD density and reduced hippocampal TG content to control values (Fig. 6G–I). Concomitantly, hippocampal ceramide levels and pro-inflammatory cytokines (IL-6, IL-1β, and TNF-α) reverted to baseline (Fig. 6J–K). Histopathology showed that butyrate mitigated inflammatory infiltrates, neuronal pyknosis, and Nissl-body loss in CA3 (Fig. 6L–M; Fig. S7H–I). Behaviorally, butyrate restored spontaneous alternation in Y-maze (working memory) and improved recognition index (RI) in novel object recognition test (episodic memory) (Fig. 6N–P). Taken together, these findings demonstrate that oral butyrate suppresses the mTORC1–ISR axis both peripherally and centrally, thereby attenuating PBAT-MPs-induced microglial lipotoxicity, neuroinflammation, and cognitive decline via the gut–brain axis. The study identifies butyrate supplementation as a readily translatable strategy to counteract gut-derived, BMP-induced neurodegeneration.

## Discussion

In the current landscape of environmental health research, microplastics (MPs) neurotoxicity has emerged as a pressing scientific concern. Whilst previous studies have largely focused on non-biodegradable MPs [33], our study provides the first systematic evidence that biodegradable PBAT-MPs impair neurological function through the gut–brain axis, and further identifies microglial lipotoxicity as a critical downstream mechanism. Oral PBAT-MPs exposure caused marked particle accumulation in intestinal tissue, accompanied by gut microbiota dysbiosis, elevated inflammation, and clear histopathological injury. Consistent with recent work demonstrating that gastrointestinal degradation of PBAT disrupts microbial homeostasis and epithelial barrier integrity [34], we further demonstrate, through FMT, that dysbiotic microbiota are sufficient to recapitulate PBAT-MPs-induced neurobehavioral deficits, thus supporting a causal gut-to-brain pathway. Mechanistically, 16S rRNA sequencing and untargeted metabolomics revealed that dysbiosis-driven perturbation of the gut–bacterial–host lipid axis elevates LD accumulation in hippocampal microglia, increases ceramide levels, and triggers maladaptive neuroimmune responses. In this way, our study extends previous descriptive observations of biodegradable MP neurotoxicity to a mechanistic level [35], identifying microglial lipotoxicity as a previously unrecognized link between intestinal PBAT-MPs exposure and neuronal dysfunction. Although astrocytes were also altered and may contribute to this process, the present study primarily focused on defining the causal role of microglial lipotoxicity, thereby providing a basis for future investigation of glial crosstalk. These findings should be interpreted in light of the study limitations discussed below.

Under physiological conditions, murine gut microbiota is dominated by fiber-degrading taxa that support epithelial integrity and immune homeostasis [36]. In this study, PBAT-MPs induced marked dysbiosis characterized by depletion of *Muribaculaceae* and *Alloprevotella*—two key SCFA-associated taxa—together with expansion of Gram-negative opportunistic pathogens, including *Escherichia*–*Shigella* and *Acinetobacter*. This shift likely reduced SCFA biosynthetic capacity whilst increasing abundance of LPS-bearing microbes, thereby promoting barrier dysfunction and amplifying peripheral and central inflammatory responses [37–39]. Multi-omics analysis further linked this microbial disruption to impaired butyrate metabolism, which emerged as a key correlate of microglial lipotoxic injury. As a major SCFA, butyrate not only supports colonic epithelial function but also exerts systemic neuroimmune effects [40]. Consistent with previous evidence that butyrate suppresses microglial inflammatory activation [41], we observed a strong inverse association between butyrate levels and indices of microglial lipotoxicity, highlighting butyrate as a potential therapeutic target in biodegradable MP-associated neurotoxicity. In FMT experiments, the protective effect of control-donor microbiota was consistent with restoration of a gut ecosystem enriched in butyrate-related functions [42]. However, these data do not demonstrate that a single donor taxon or metabolite solely accounts for the rescue phenotype. Rather, FMT is more appropriately interpreted as reconstituting a broader ecological and metabolic environment favorable for butyrate production and inflammatory restraint. Because we did not perform donor metagenomic/functional profiling or transplantation with defined microbial consortia, we avoid stronger causal claims regarding specific butyrate-producing lineages [43, 44]. Thus, our FMT results support a community-level, butyrate-favorable mechanism, in agreement with the metabolomics and butyrate supplementation data, whilst the contribution of individual strains remains to be resolved.

At the molecular level, our study identifies mTORC1 as an upstream regulator of ISR signaling in PBAT-MPs-induced microglial lipotoxicity along the gut–brain axis. Gain-of-function experiments showed that butyrate exerts neuroprotective effects by suppressing the mTORC1–ISR pathway. In the setting of gut-derived neuroinflammation, ISR activation robustly promoted transcription of LD biogenesis genes, leading to excessive neutral-lipid accumulation in microglia, whereas pharmacological ISR blockade (with ISRIB) largely reversed the LD burden evoked by inflammatory stimuli, confirming the causal role of ISR in lipotoxic injury. Importantly, butyrate—a gut microbiota-derived metabolite—markedly restored lipid homeostasis, highlighting the profound influence of intestinal ecological balance on brain metabolism. Mechanistically, butyrate directly targeted mTORC1, residing genetically upstream of ISR. Overexpression of Rptor (a core component of mTORC1) in HMC-3 microglia blunted butyrate’s capacity to suppress ISR signaling and to restrain LD formation, establishing mTORC1 as the obligatory node in butyrate-mediated neuroprotection. It is noteworthy that hierarchical interplay between mTORC1 and ISR remains contentious. Some reports indicate that ISR can attenuate mTORC1 activity [45], whereas others, in line with present data, show that mTORC1 activation fuels ISR signaling [46]. Such contextual directionality likely reflects cell type-specific wiring, stressor identity, or microenvironmental cues, implicating a putative bidirectional regulatory circuit. In this study, in vivo butyrate supplementation experiments were intended to support the pathophysiological relevance of the mTORC1–ISR-associated microglial lipotoxicity axis in the hippocampus, rather than to establish strict microglia-autonomous regulation or definitive pathway hierarchy in vivo. Given the pleiotropic actions of butyrate across the gut–brain axis—including effects on intestinal barrier, vascular interface, peripheral immunity, and multiple CNS-resident cell types [47, 48]—we therefore define pathway hierarchy primarily on the basis of our controlled in vitro data, whilst regarding in vivo results as complementary evidence of disease relevance.

Butyrate, a gut microbiota-derived metabolite generated by fermentation of dietary fiber, has emerged as a promising therapeutic candidate for neurodegenerative diseases. Previous studies have shown that oral butyrate supplementation ameliorates neuropathology in Parkinson’s disease (PD) and Alzheimer’s disease (AD) models [49]. In MPTP-treated mice, butyrate reduced the *Firmicutes/Bacteroidetes* ratio and increased beneficial SCFA producers (e.g., *Ruminococcaceae*, *Lachnospiraceae*), correlating with preserved nigral dopaminergic neurons [50]. Likewise, 12-week butyrate administration in 5×FAD mice reduced cerebral Aβ plaque load and restored cognitive function and memory performance [51]. In the present study, we extend these findings by demonstrating that butyrate delivered via drinking water efficiently counteracts PBAT-MPs-induced microglial lipotoxicity and neurodegeneration. Relative to PBAT-MPs exposure alone, butyrate selectively silenced the mTORC1–ISR axis, blocked LD biogenesis, and alleviated lipotoxic neuro-inflammation and neuronal damage. To our knowledge, this is the first intervention to specifically target microglial lipid-metabolic dysfunction evoked by butyrate depletion, providing pre-clinical proof-of-concept for repurposing butyrate against MPs-triggered neurotoxicity and broadening its therapeutic scope in neurodegenerative diseases. The use of five mice per group reflected a balance between experimental control and data reliability under highly standardized conditions, including same-cage housing, age- and sex-matching, and a single experimenter. This sample size is consistent with prior mechanistic studies and the 3R principles [52, 53], although the relatively small cohort remains a limitation and should be expanded in future work. In addition, this study was conducted exclusively in male mice. We adopted a male-only design in this preliminary mechanistic investigation to reduce endocrine-related variability associated with the estrous cycle and estrogen, which can modulate microglial activation and neuroinflammatory responses, thereby facilitating more stable identification of gut–brain inflammatory signaling and glial lipotoxic mechanisms following PBAT-MPs exposure. However, this approach inevitably limits the generalizability of our findings, as sex may influence gut microbiota composition, immune response patterns, and central nervous system inflammatory processes. Thus, our study does not address potential female-specific pathological or protective responses to PBAT-MPs. Future studies should include larger cohorts of both sexes and further clarify sex-dependent toxicological responses, as well as the coordinated roles of astrocytes and microglia in PBAT-MPs-induced neural injury.

## Conclusion

Integrating in vivo and in vitro models, we identify butyrate as a key upstream suppressor of PBAT-MPs-induced microglial lipotoxicity. Mechanistically, butyrate maintains mTORC1–ISR homeostasis, thereby limiting LD biogenesis, neuroinflammation, and neuronal injury. In contrast, PBAT-MPs exposure depletes butyrate-producing taxa, reduces butyrate availability, over-activates mTORC1–ISR signaling, and accelerates neurodegeneration. These findings define a previously unrecognized microbiota–butyrate–mTORC1–ISR axis as a central driver of PBAT-MPs-induced brain injury, and suggest that restoring butyrate signaling or targeting the downstream mTORC1–ISR pathway may represent a translatable strategy for preventing BMP-related neurodegeneration. Notably, both astrocytes and microglia were activated in this model, indicating potential glial crosstalk. Future studies using cell type-specific approaches are needed to clarify their interaction and its contribution to PBAT-MPs-induced neuroinflammation.

## Supplementary Information

Supplementary Material 1.

Supplementary Material 2.

## Data Availability

All data supporting the ﬁndings of this study are available from the corresponding author upon reasonable request.
