# Supplementary material for: Butyrate-producing gut bacteria restrain PBAT microplastic-triggered brain microglial lipotoxicity via a microbiota–butyrate–mTORC1–ISR relay along the gut–brain axis
Source: J Neuroinflammation. 2026 May 13;23:233. doi: 10.1186/s12974-026-03869-1 (PMC13343657; doi:10.1186/s12974-026-03869-1)
Supplement: Supplementary file 1 — Supplementary Material 1. [file 12974_2026_3869_MOESM1_ESM.docx]

**Original WB bands**


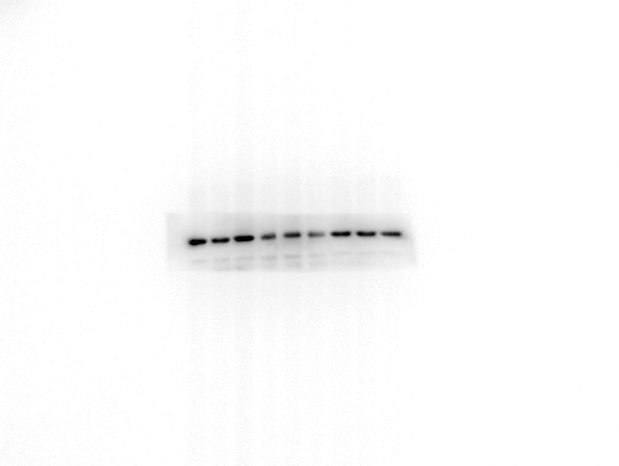

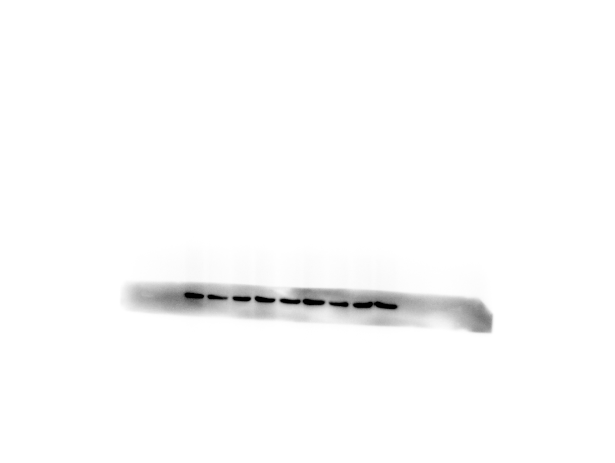

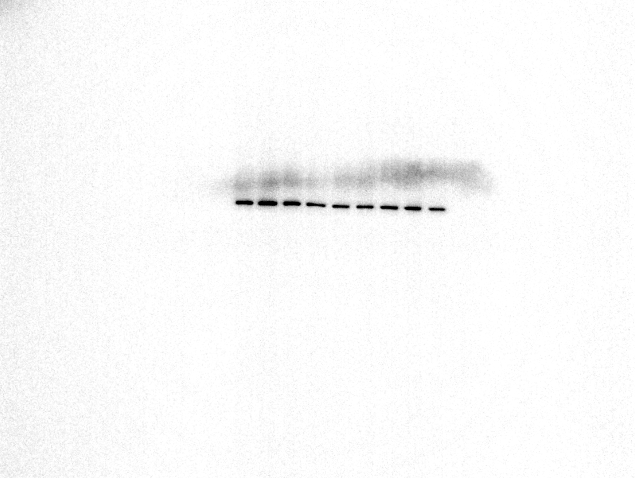

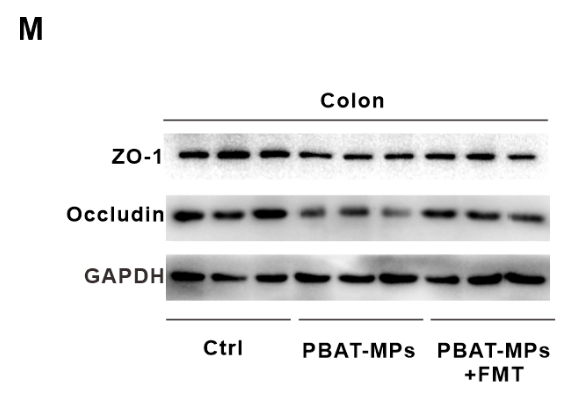
**Fig. 1.**

**Fig. 1. GAPDH**

**Fig. 1. Occludin**

**Fig. 1. ZO-1**


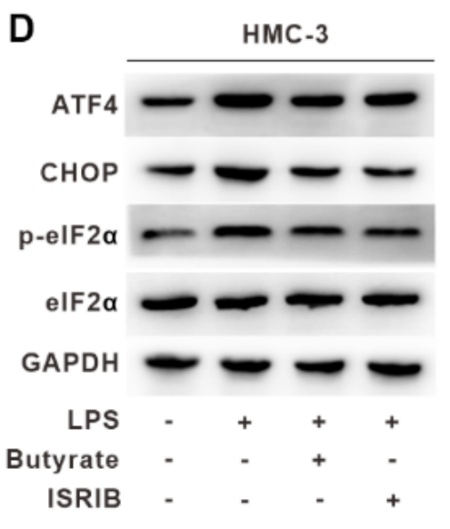
**Fig. 4.**


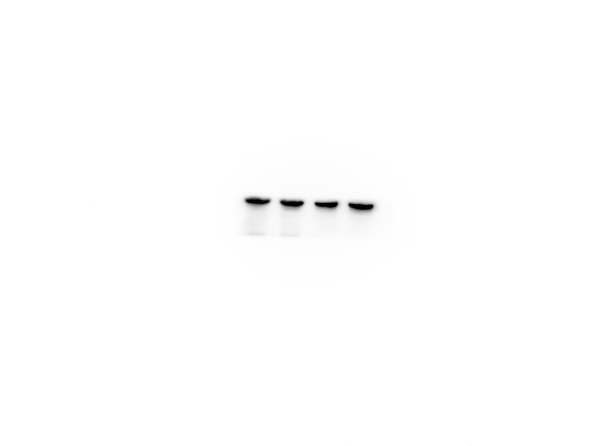

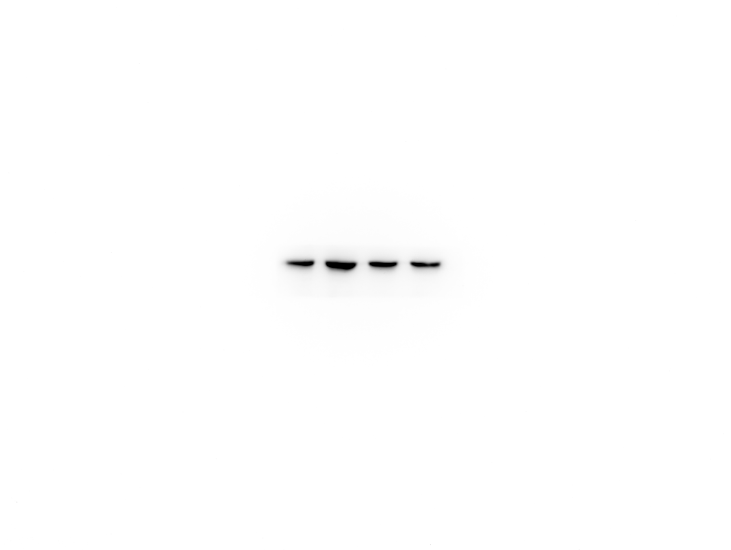

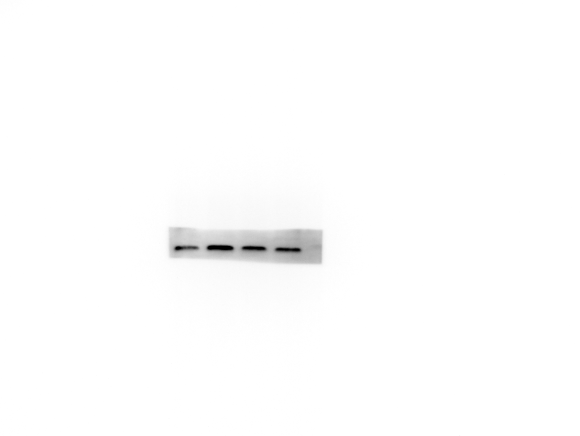

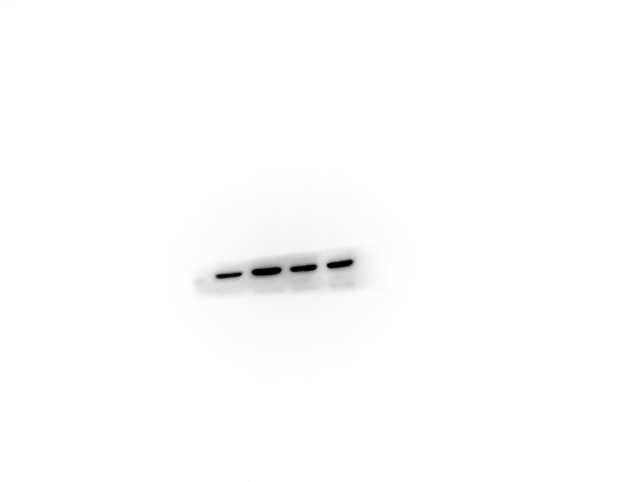

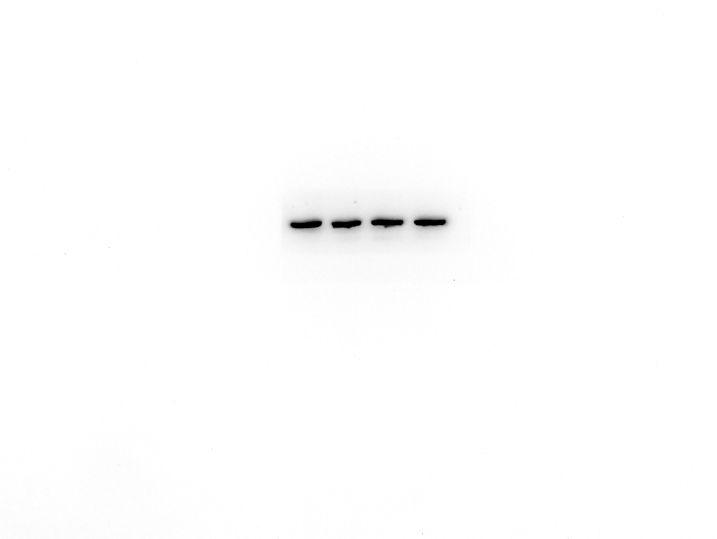


**Fig. 4. GAPDH**

**Fig. 4. eIF2a**

**Fig. 4. p-eIF2a**

**Fig. 4. CHOP**

**Fig. 4. ATF4**


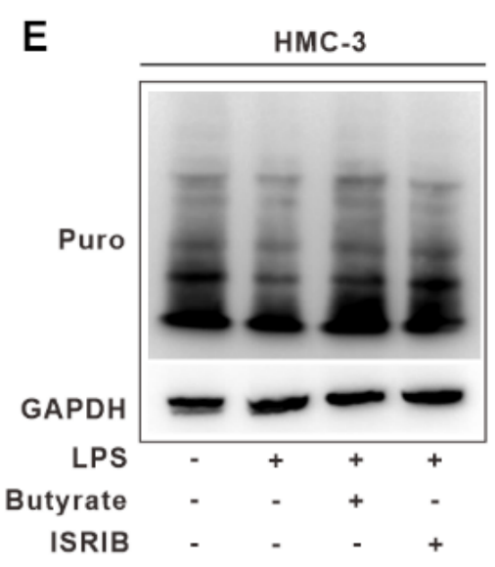
**Fig. 4.**


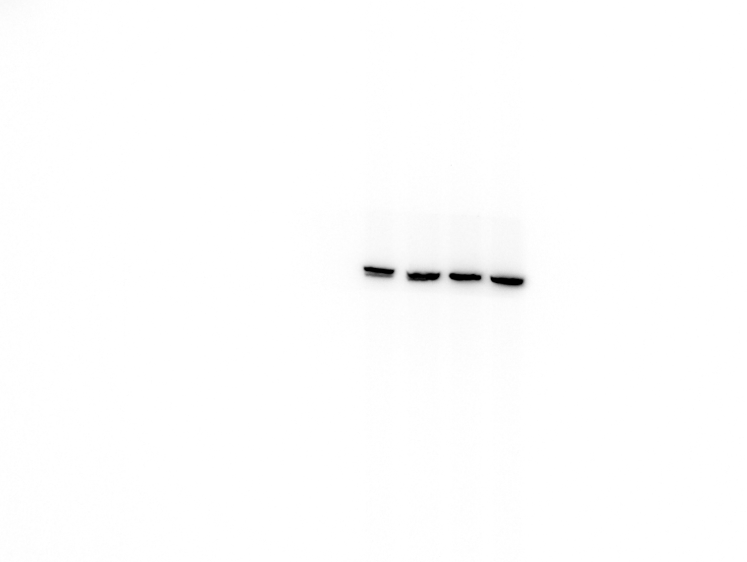

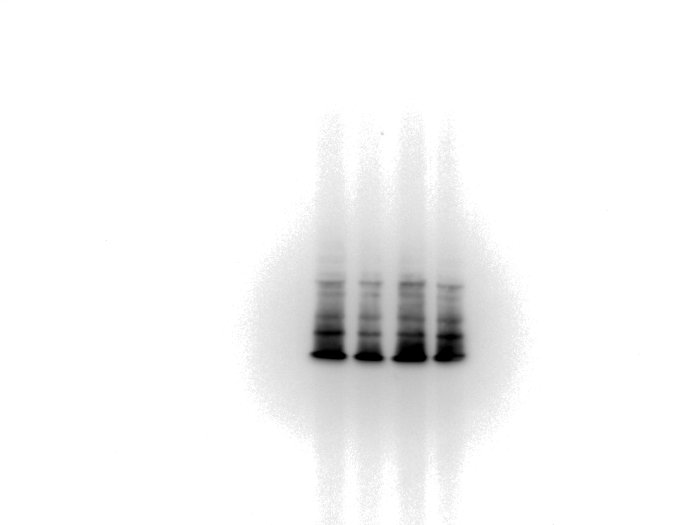


**Fig. 4. GAPDH**

**Fig. 4. Puro**


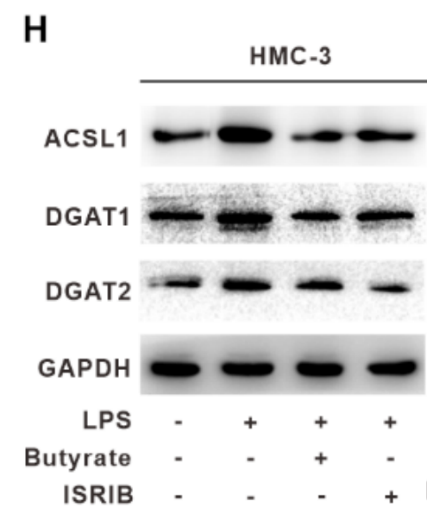
**Fig. 4.**


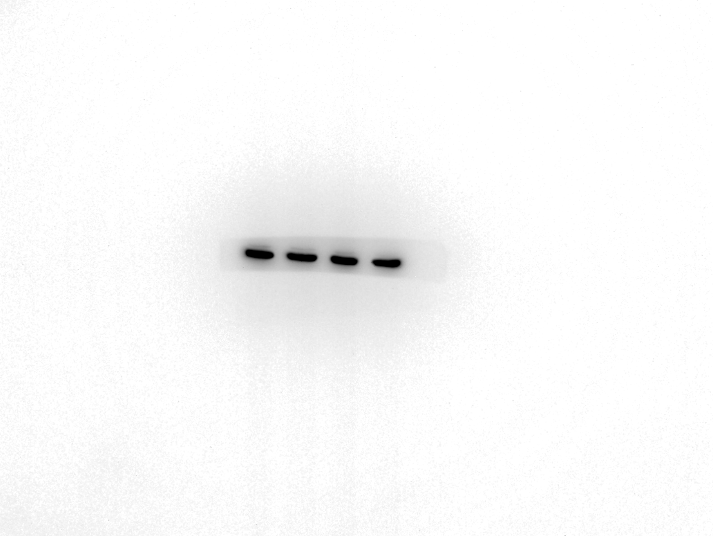

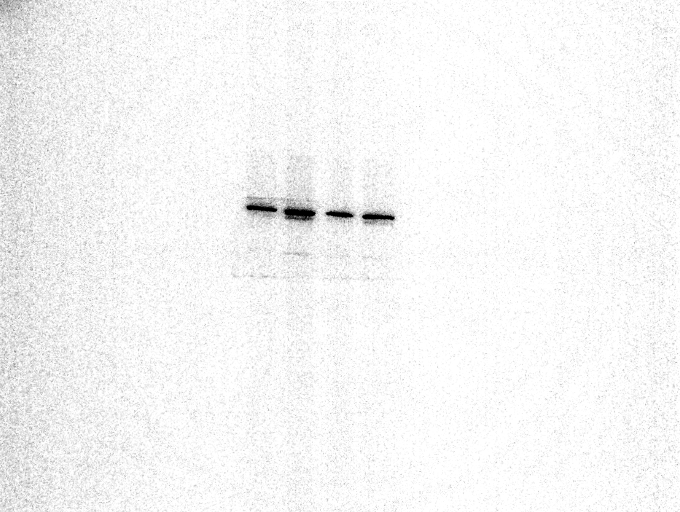

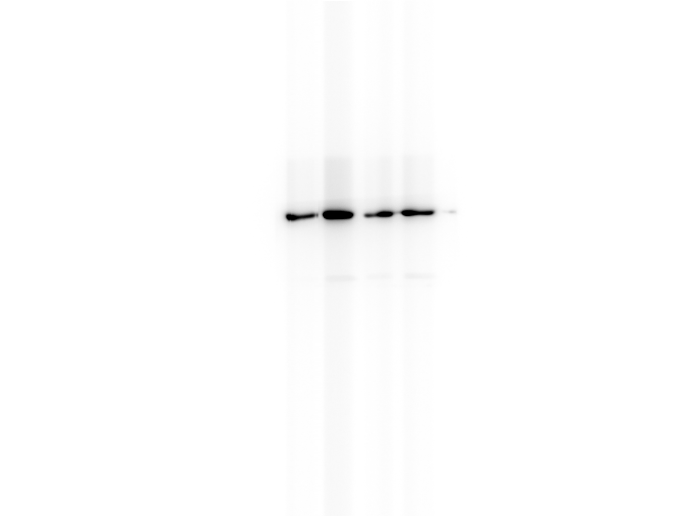

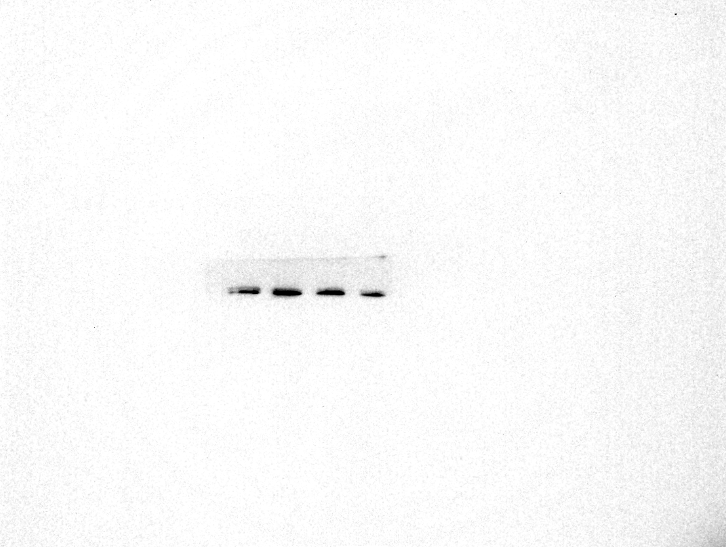


**Fig. 4. DGAT2**

**Fig. 4. GAPDH**

**Fig. 4. DGAT1**

**Fig. 4. ACSL1**


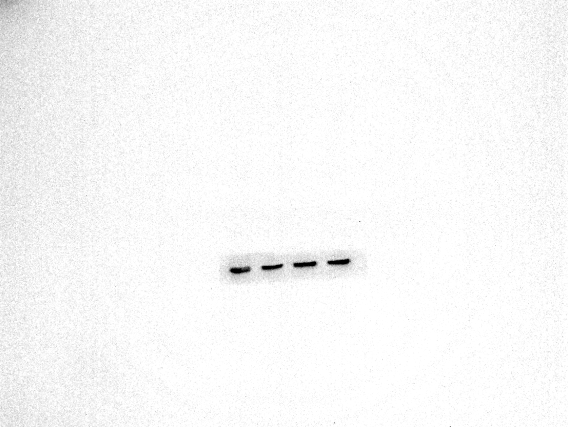

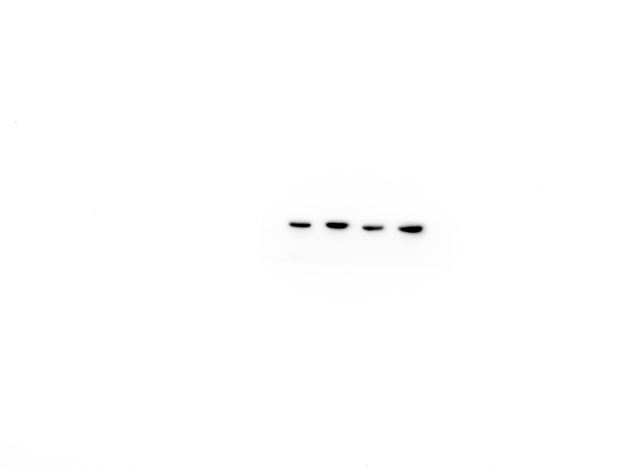

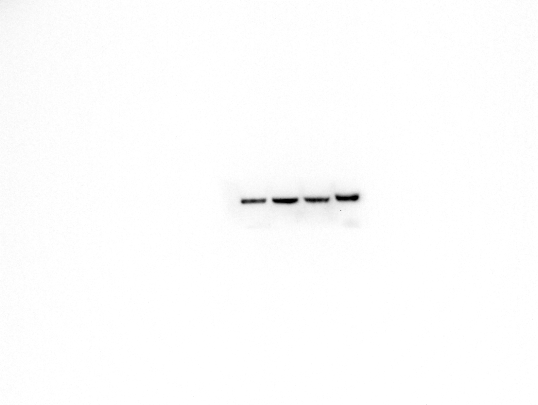

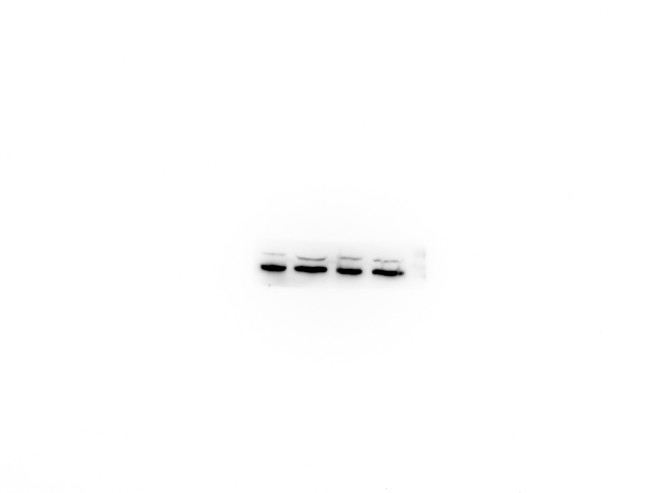

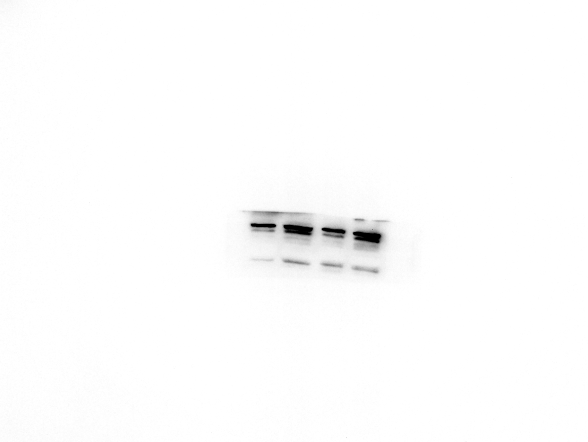

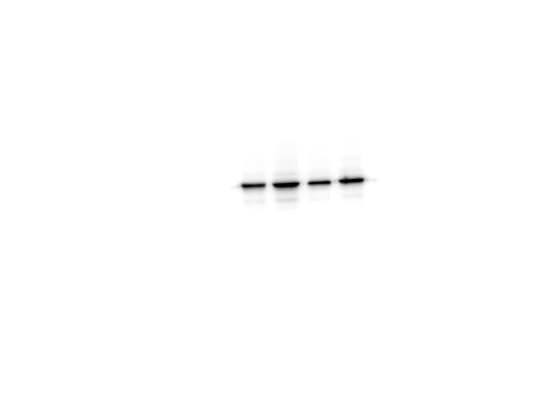

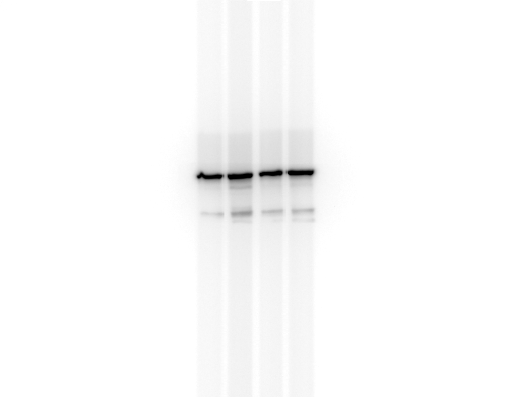

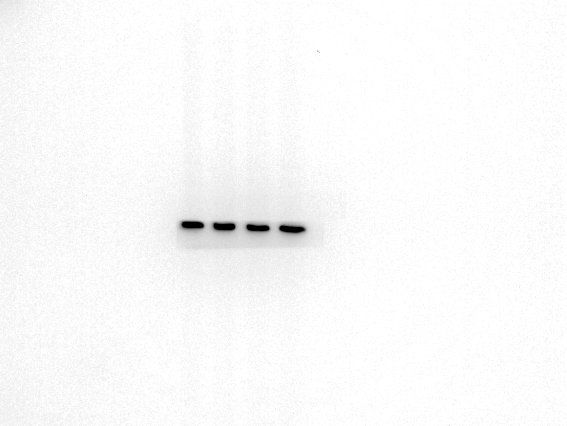

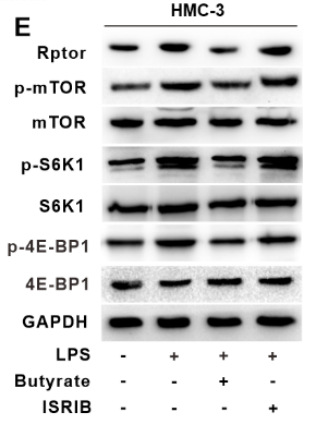
**Fig. 5.**

**Fig. 5. GAPDH**

**Fig. 5. 4E-BP1**

**Fig. 5. S6K1**

**Fig. 5. p-4E-BP1**

**Fig. 5. p-S6K1**

**Fig. 5. mTOR**

**Fig. 5. p-mTOR**

**Fig. 5. Rptor**

**Fig. 5.**


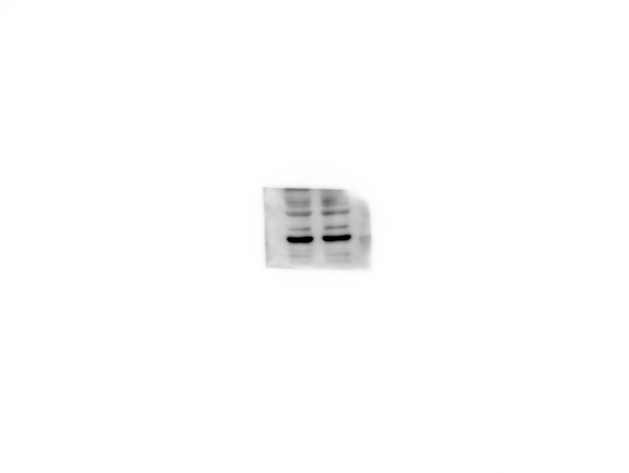

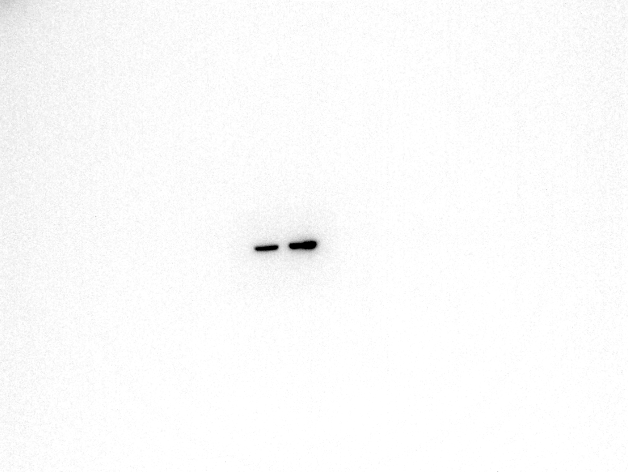

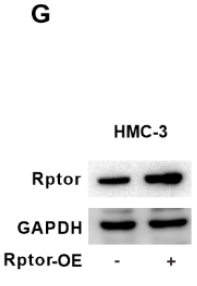


**Fig. 5. GAPDH**

**Fig. 5. Rptor**

**Fig. S4.**


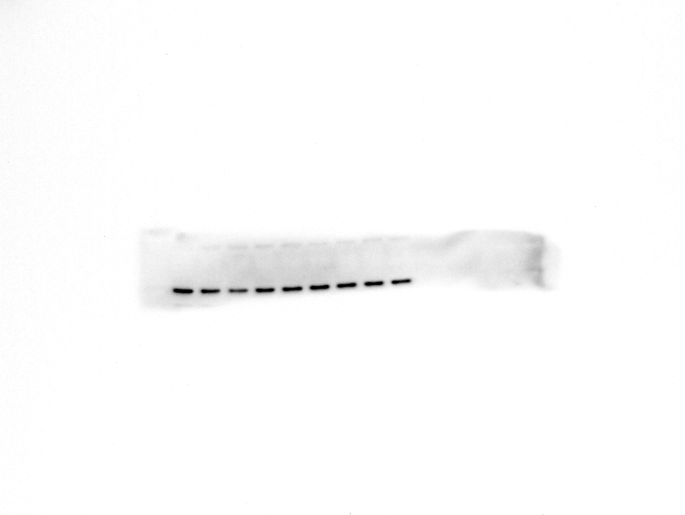

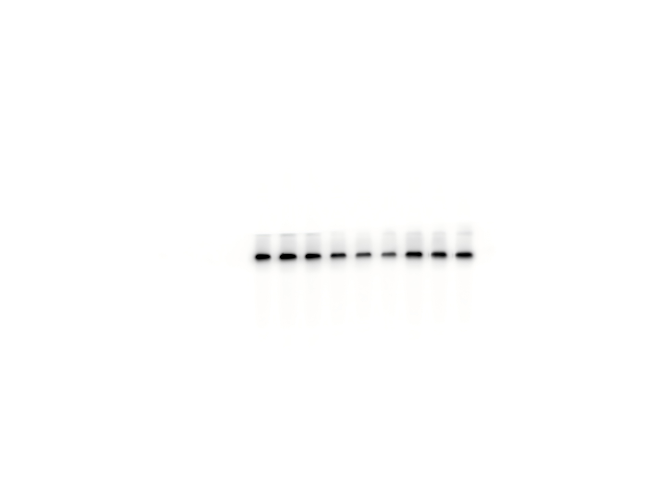

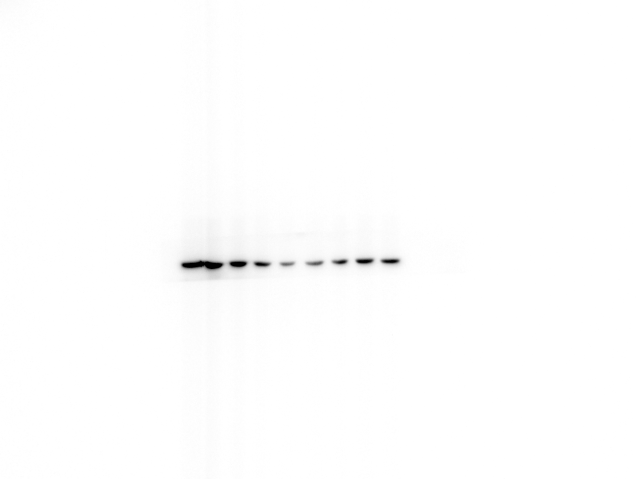

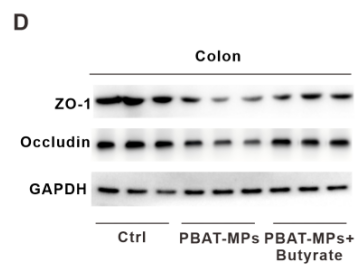


**Fig. S4. GAPDH**

**Fig. S4. Occludin**

**Fig. S4. ZO-1**

**Fig. 6.**


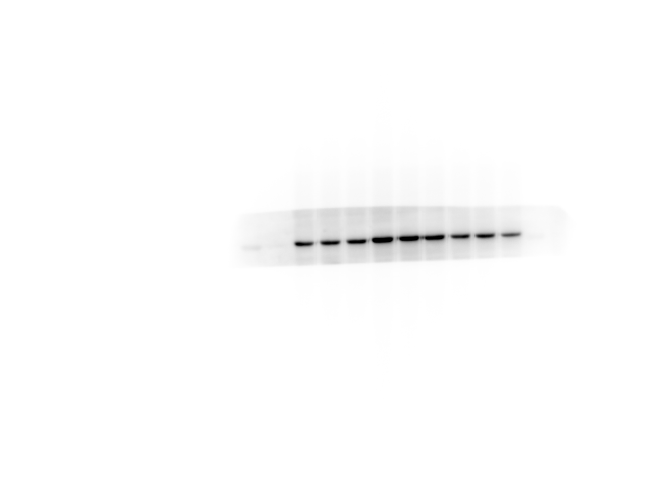

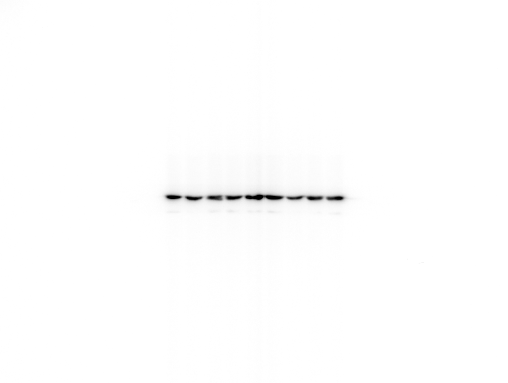

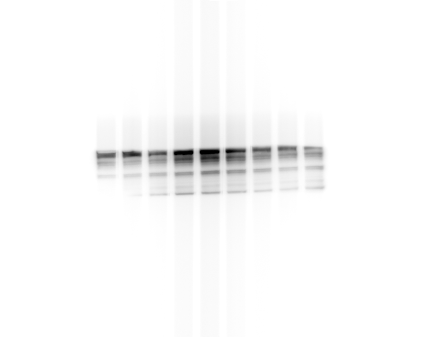

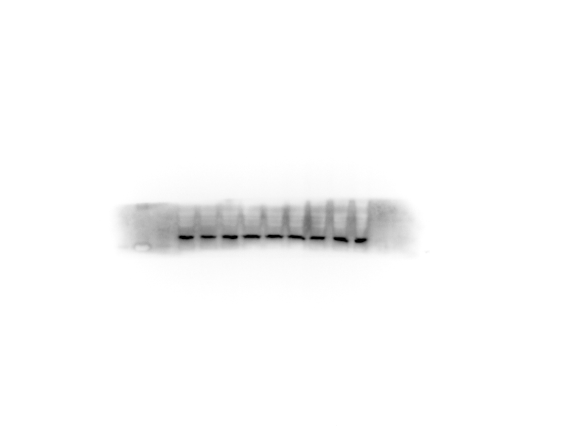

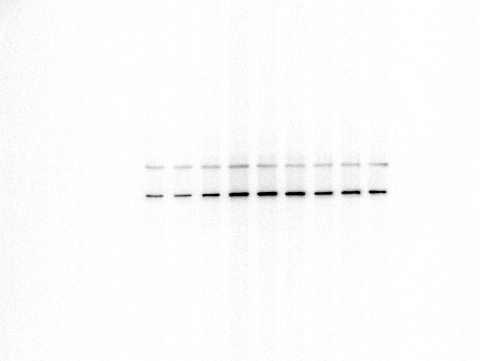

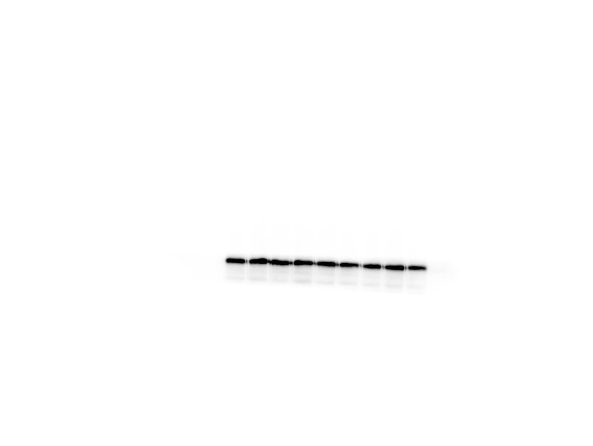

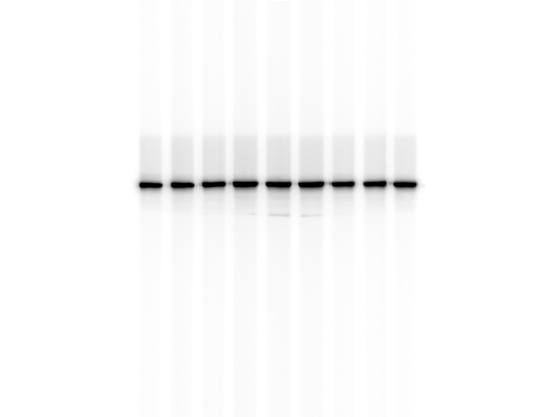

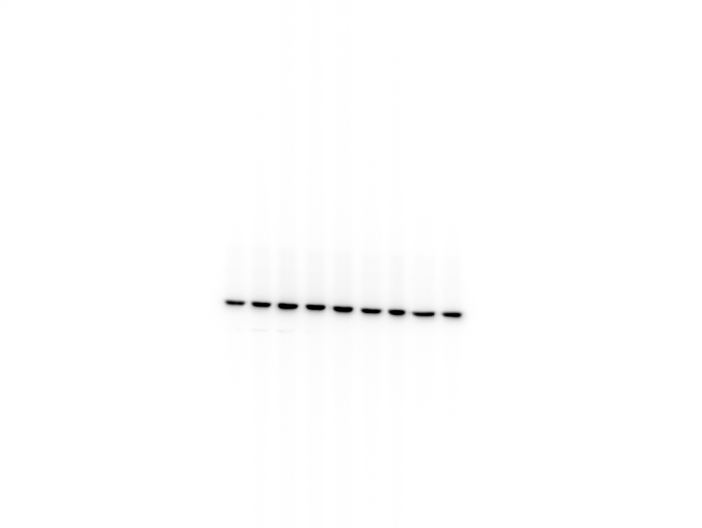

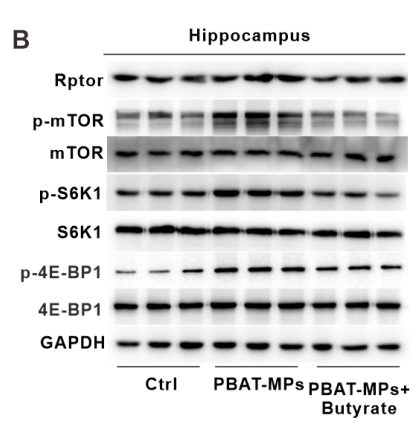


**Fig. 6. 4E-BP1**

**Fig. 6. GAPDH**

**Fig. 6. p-4E-BP1**

**Fig. 6. S6K1**

**Fig. 6. p-S6K1**

**Fig. 6. mTOR**

**Fig. 6. p-mTOR**

**Fig. 6. Rptor**

**Fig. 6.**


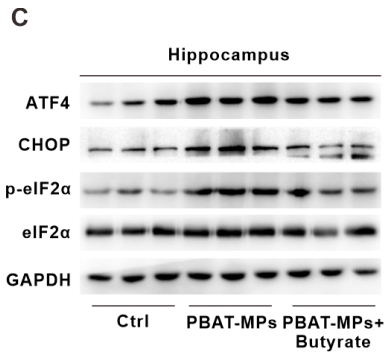


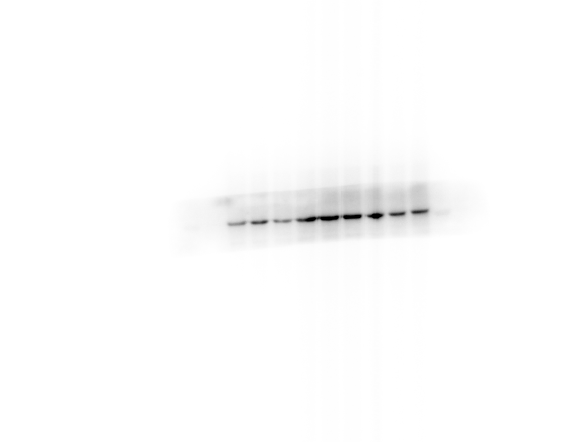

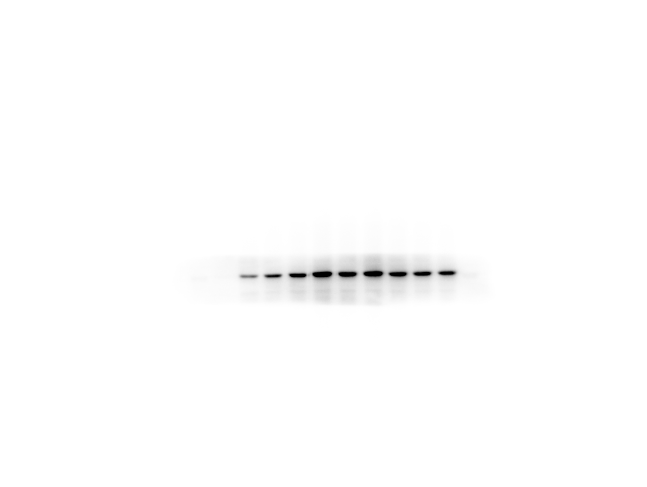

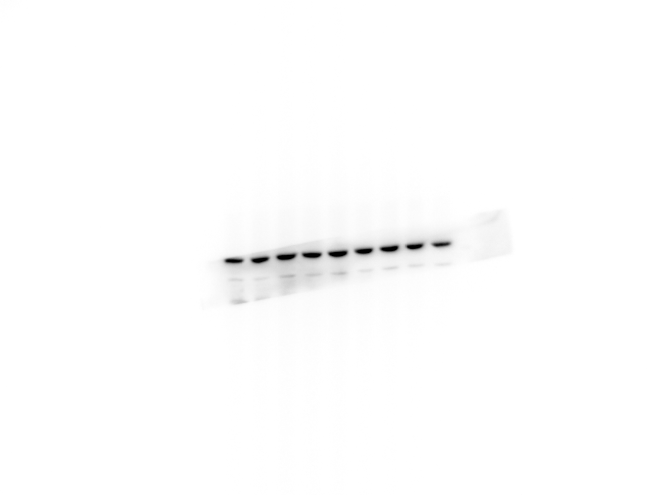

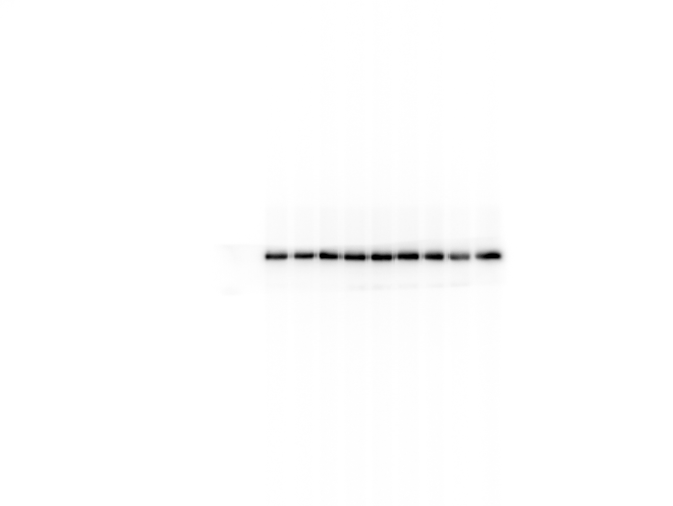

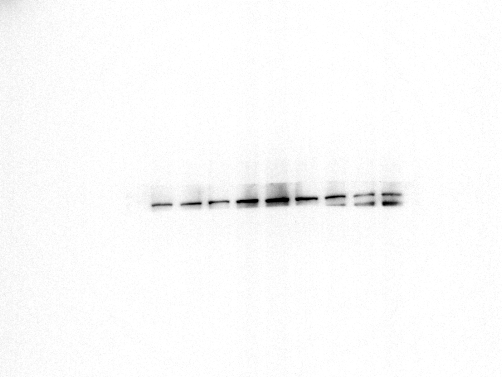


**Fig. 6. GAPDH**

**Fig. 6. p-eIF2a**

**Fig. 6. eIF2a**

**Fig. 6. CHOP**

**Fig. 6. ATF4**

**Fig. 6.**


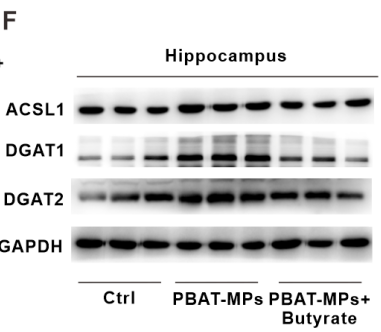


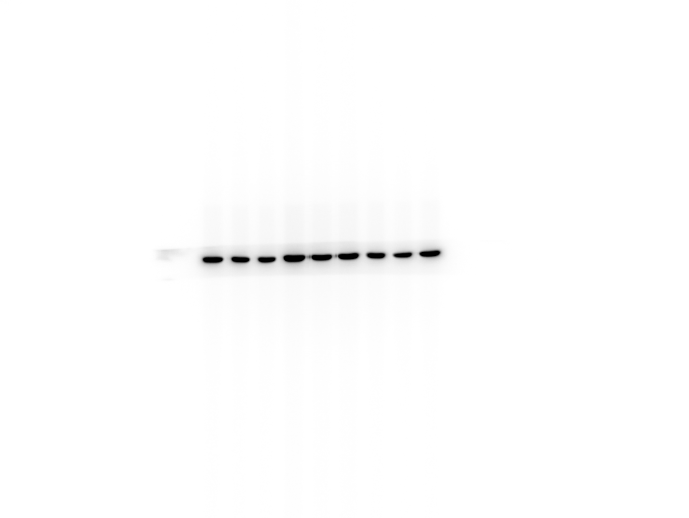

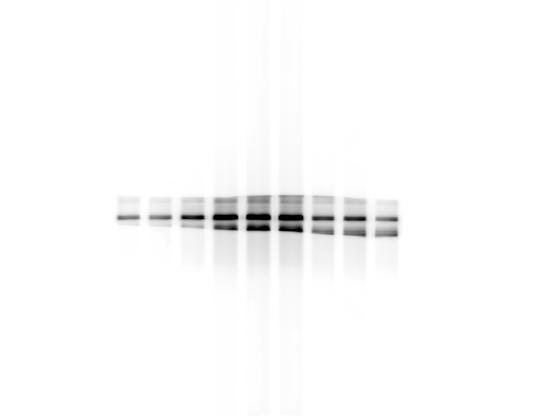

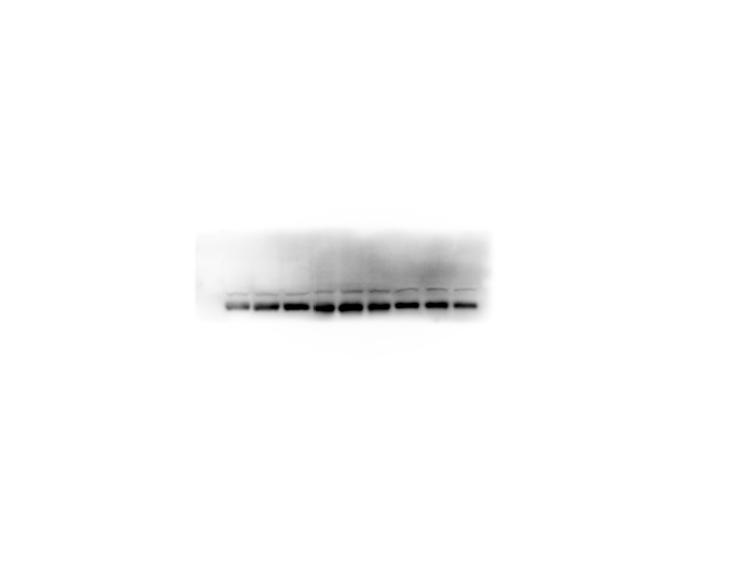

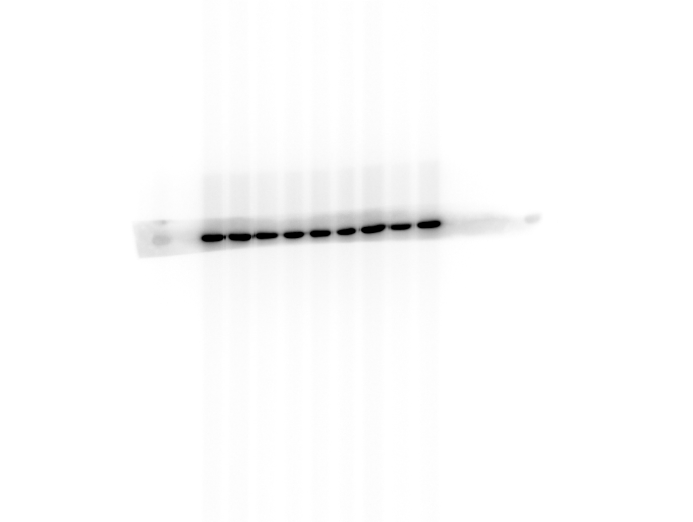


**Fig. 6. DGAT2**

**Fig. 6. GAPDH**

**Fig. 6. DGAT1**

**Fig. 6. ACSL1**
