# Supplementary material for: Butyrate-producing gut bacteria restrain PBAT microplastic-triggered brain microglial lipotoxicity via a microbiota–butyrate–mTORC1–ISR relay along the gut–brain axis
Source: J Neuroinflammation. 2026 May 13;23:233. doi: 10.1186/s12974-026-03869-1 (PMC13343657; doi:10.1186/s12974-026-03869-1)
Supplement: Supplementary file 2 — Supplementary Material 2. [file 12974_2026_3869_MOESM2_ESM.docx]

**Butyrate-producing gut bacteria restrain PBAT microplastic-triggered brain microglial lipotoxicity via a microbiota–butyrate–mTORC1–ISR relay along the gut–brain axis**

Ming-Zhu Wang ^1,#^, Ze-Bang Du ^1,#^, Wen-Qi Xu ^1,#^, Yu-Han Xie ^1^, Lei-Lei Wang ^1^, Xin-Xin He ^1^, Yu-Han Wang ^2^, Han-Ying Zheng ^1^, You-Liang Yao ^1^, Ya-Bin Song ^2,*^, Zhong-Ning Lin ^1,^*, Yu-Chun Lin ^1,^*.

1. State Key Laboratory of Vaccines for Infectious Diseases, Xiang An Biomedicine Laboratory, Xiang'an Hospital of Xiamen University, National Innovation Platform for Industry-Education Integration in Vaccine Research, School of Public Health, Xiamen University, Xiamen, 361102, China.

2. Department of Neurology, Xiang'an Hospital of Xiamen University, Xiamen, Fujian, China.

^#^ These authors contributed equally to this work.

* Corresponding Author: State Key Laboratory of Vaccines for Infectious Diseases, Xiang An Biomedicine Laboratory, Xiang'an Hospital of Xiamen University, School of Public Health, Xiamen University, Xiang'an South Road, Xiamen, 361102, China.

Tel: +86 592 2880615; Fax: +86 592 2881578

E-mail: songyabin1977@126.com (Ya-Bin Song), linzhn@xmu.edu.cn (Zhong-Ning Lin), linych@xmu.edu.cn (Yu-Chun Lin)**Supplementary figures and legends**

**
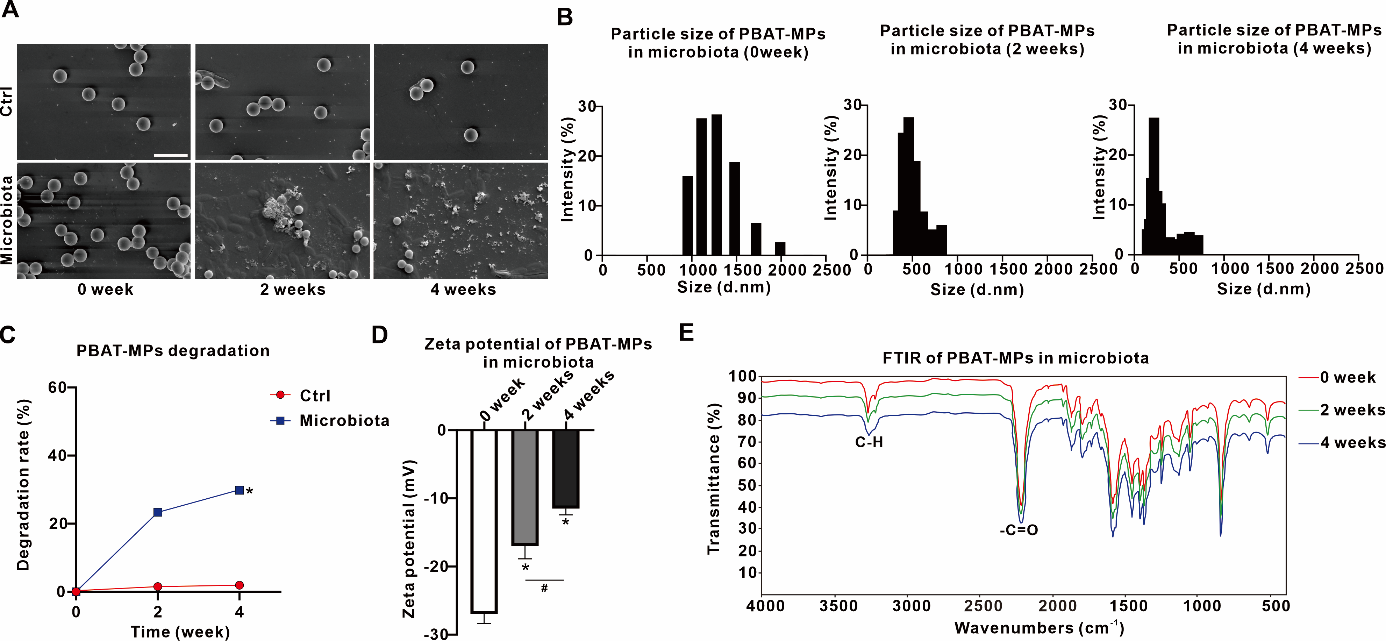
****Fig. S1. Physicochemical characterization of PBAT-MPs.** Analyses were performed fortnightly throughout a 4-week *in vitro* simulated gastrointestinal digestion with gut microbiota. (A) Representative transmission electron microscopy (TEM) images showing particle morphology and size; scale bar, 3 μm. (B) Hydrodynamic diameter measured by dynamic light scattering (DLS). (C) *In vitro* degradation rate by gut microbiota. (D) Zeta potential measured by DLS. (E) Fourier-transform infrared (FTIR) spectra for compositional verification. * *P* < 0.05 versus control.

**
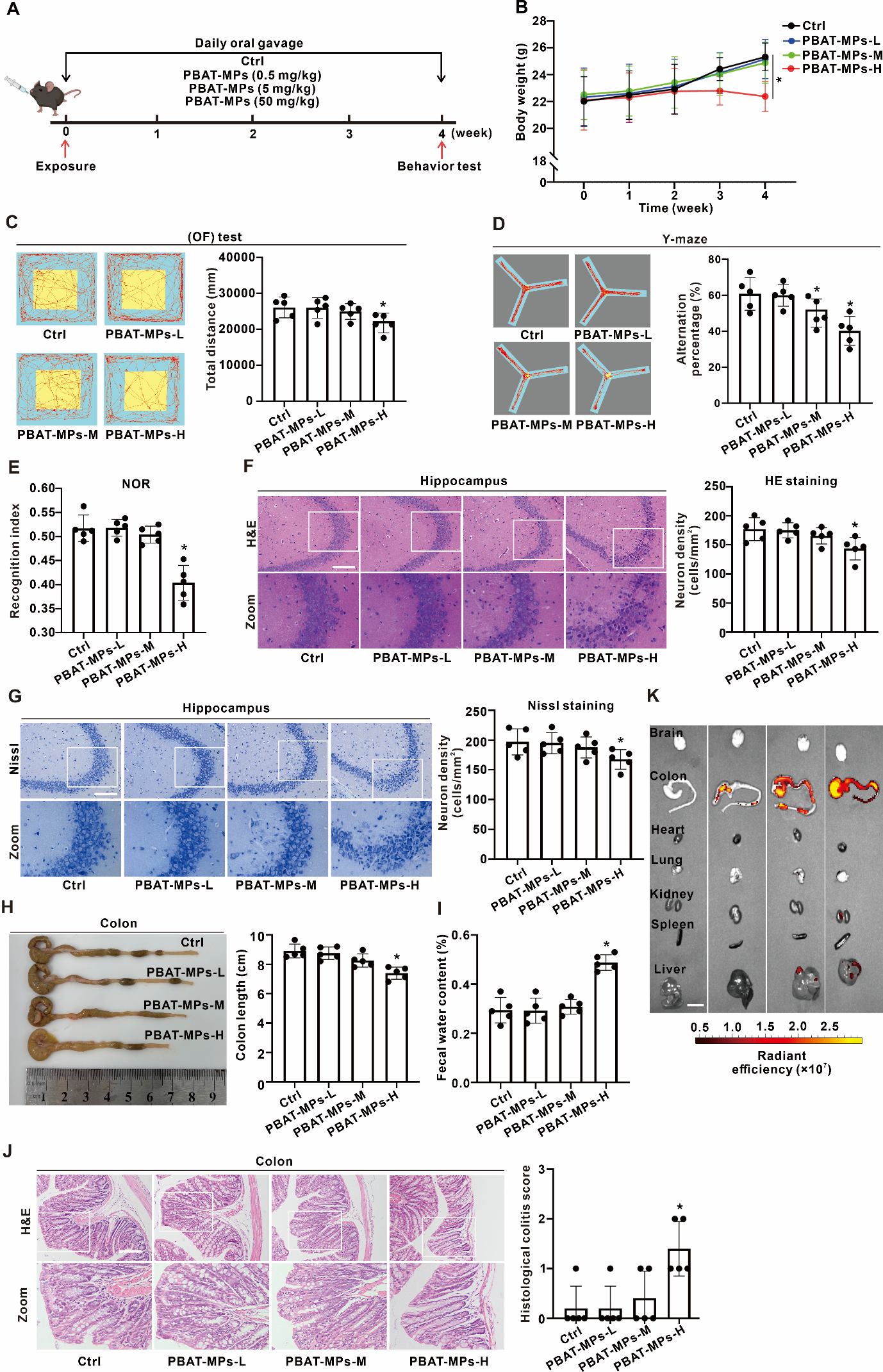
****Fig. S2.** **PBAT-MPs induce dose-dependent behavioral impairment, hippocampal neuronal injury, and colonic abnormalities.** C57BL/6 mice received daily oral gavage of PBAT-MPs at 0.5 mg kg⁻¹ (PBAT-MPs-L), 5 mg kg⁻¹ (PBAT-MPs-M), or 50 mg kg⁻¹ (PBAT-MPs-H) for 4 weeks. n = 5 per group. (A) Flow chart of animal experiment design. (B) Body weight during treatment; n = 5. (C) Representative track images in open-field test (left); total distance (right); n = 5. (D) Representative track images in Y-maze test (left); spontaneous alternation percentage (right); n = 5. (E) Novel object recognition (NOR) test showing the recognition index; n = 5. (F) Representative H&E-stained images of hippocampal CA3 (left); scale bar, 100 μm. Quantification of neuronal density (right). (G) Representative Nissl-stained images of hippocampal CA3 (left); scale bar, 100 μm. Quantification of neuronal density (right). (H) Representative images of colon tissue (left); quantification of colon length (right). (I) Water percentage of fecal pellets. (J) Representative H&E-stained images of colon tissue (left); scale bar, 100 μm. Quantification of histological colitis scores (right). (K) Representative ex vivo fluorescence images showing PBAT-MPs accumulation in major organs; scale bar, 1 cm. * *P* < 0.05 versus control; # *P* < 0.05 versus corresponding group.

**
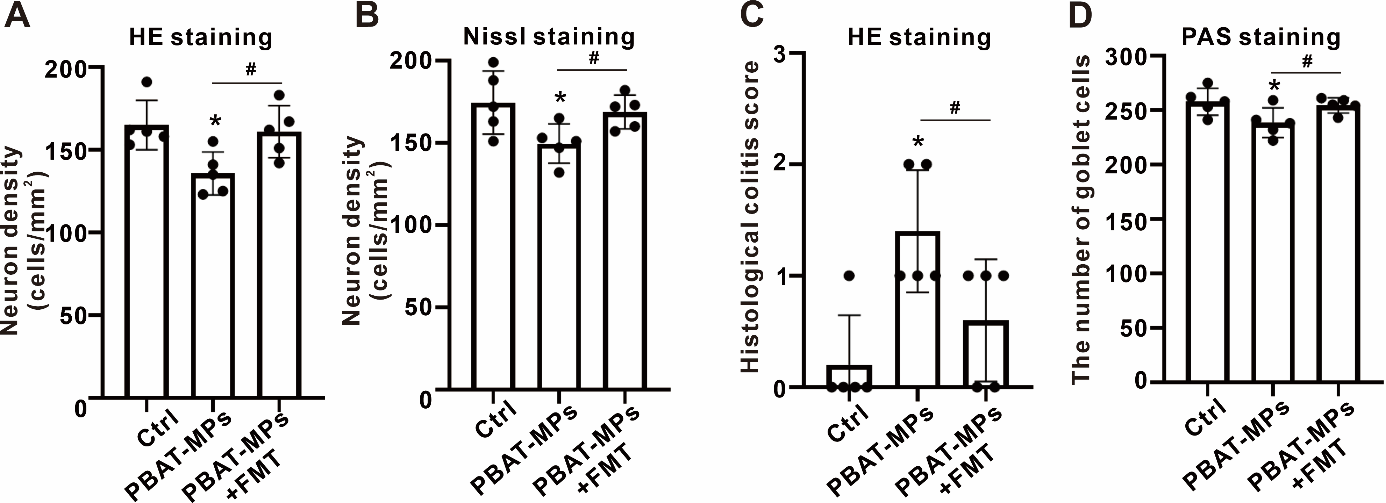
****Fig. S3.** **Quantitative analysis of brain and intestinal histopathology.** C57BL/6 mice received daily oral gavage of PBAT-MPs (50 mg kg⁻¹) for 4 weeks, followed by treatment with or without FMT for 2 weeks. n = 5 per group. (A–B) Hippocampal neuronal density from H&E (A) and Nissl (B) staining. (C–D) Colonic histopathology: H&E-based colitis scores (C) and PAS-based goblet cell counts (D). * *P* < 0.05 versus control; # *P* < 0.05 versus corresponding group.

**
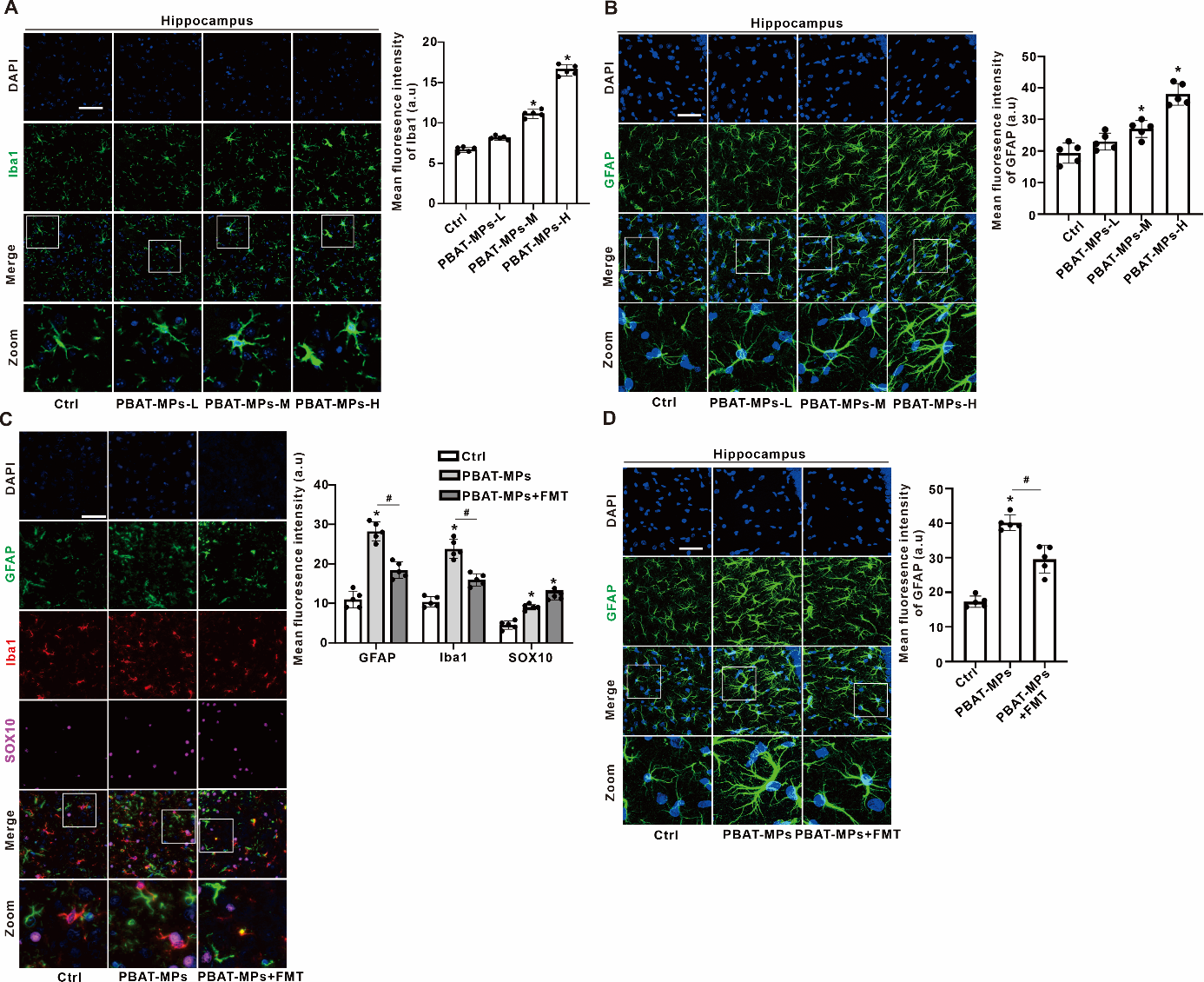
Fig. S4.** **PBAT-MPs exposure induces glial activation in the hippocampus, with partial reversal by FMT.** (A–B) C57BL/6 mice received daily oral gavage of PBAT-MPs at 0.5 mg kg⁻¹ (PBAT-MPs-L), 5 mg kg⁻¹ (PBAT-MPs-M), or 50 mg kg⁻¹ (PBAT-MPs-H) for 4 weeks. n = 5 per group. (A) Representative IF images of sections stained for Iba1 (green) and DAPI (blue) in hippocampal CA3 (left); scale bar, 50 μm. Quantification of Iba1 mean fluorescence intensity (right). (B) Representative IF images of sections stained for GFAP (green) and DAPI (blue) in hippocampal CA3 (left); scale bar, 50 μm. Quantification of GFAP mean fluorescence intensity (right). (C–D) C57BL/6 mice received daily oral gavage of PBAT-MPs (50 mg kg⁻¹) for 4 weeks, followed by treatment with or without FMT for 2 weeks. n = 5 per group. (C) Representative multiplex IF images of sections co-stained for GFAP (astrocytes, green), Iba1 (microglia, red), SOX10 (oligodendroglial lineage cells, magenta), and DAPI (nuclei, blue) in hippocampal CA3 (left); scale bar, 50 μm. Quantification of mean fluorescence intensity (right). (D) Representative IF images of sections stained for GFAP (green) and DAPI (blue) in hippocampal CA3 (left); scale bar, 50 μm. Quantification of GFAP mean fluorescence intensity (right). * *P* < 0.05 versus control; # *P* < 0.05 versus corresponding group.

**
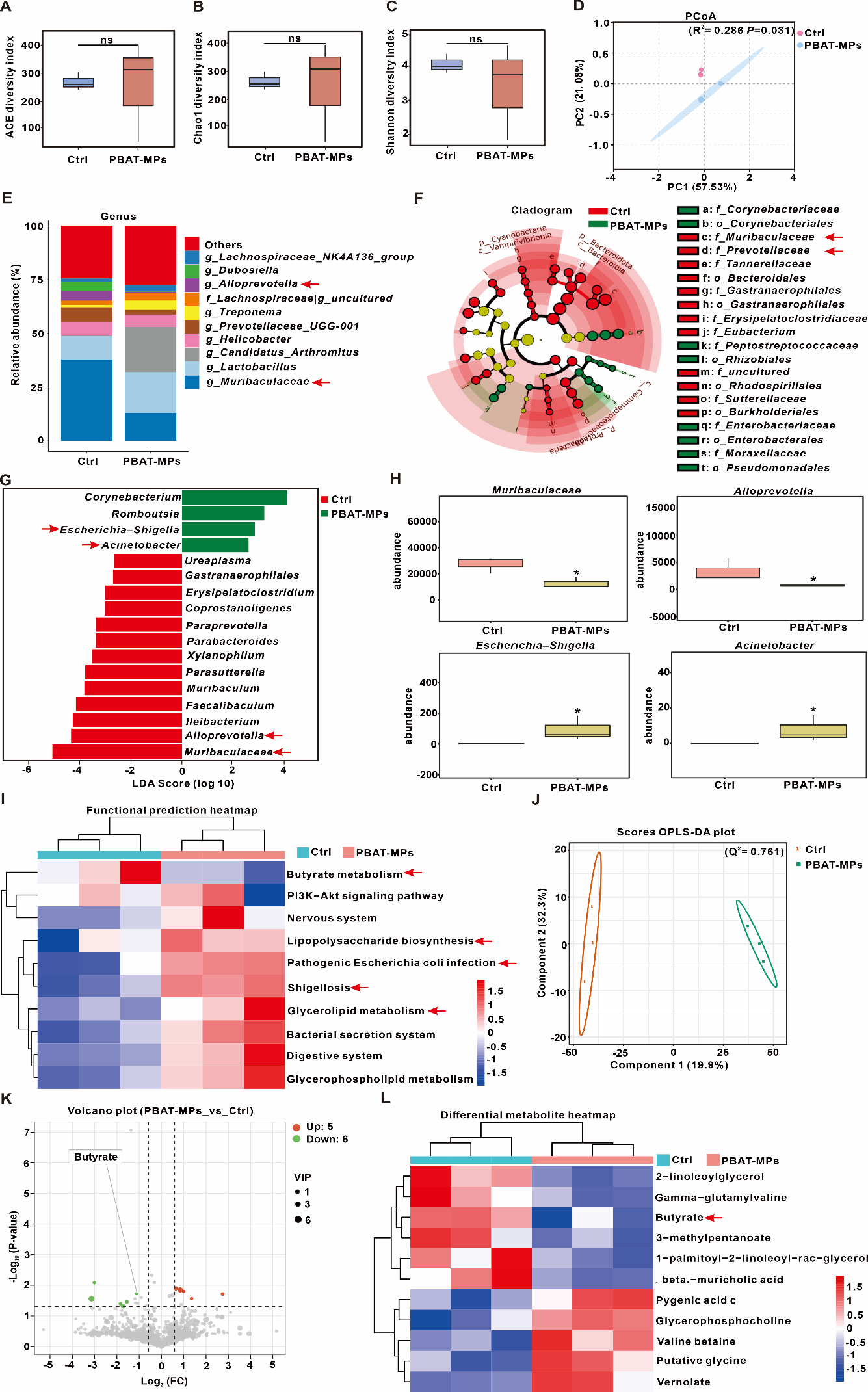
**

**Fig. S5.** **PBAT-MPs exposure induces alterations in gut microbiota and metabolites.** (A–C) Alpha diversity indices: ACE (A), Chao1 (B), and Shannon (C). (D) Beta diversity analyzed by principal coordinate analysis (PcoA). (E) Gut microbiota composition at genus level. (F) Phylogenetic tree; dot size denotes relative abundance. (G) LEfSe comparison of differentially abundant taxa. (H) Inter-group differences in *Muribaculaceae*, *Alloprevotella*, *Escherichia*–*Shigella*, and *Acinetobacter*. (I) KEGG pathway prediction of differential microbial functions. (J) OPLS-DA score plot for microbial metabolites. (K) Volcano plot of differential microbial metabolites. (L) Heat map of differential microbial metabolites. * *P* < 0.05 versus control.

**
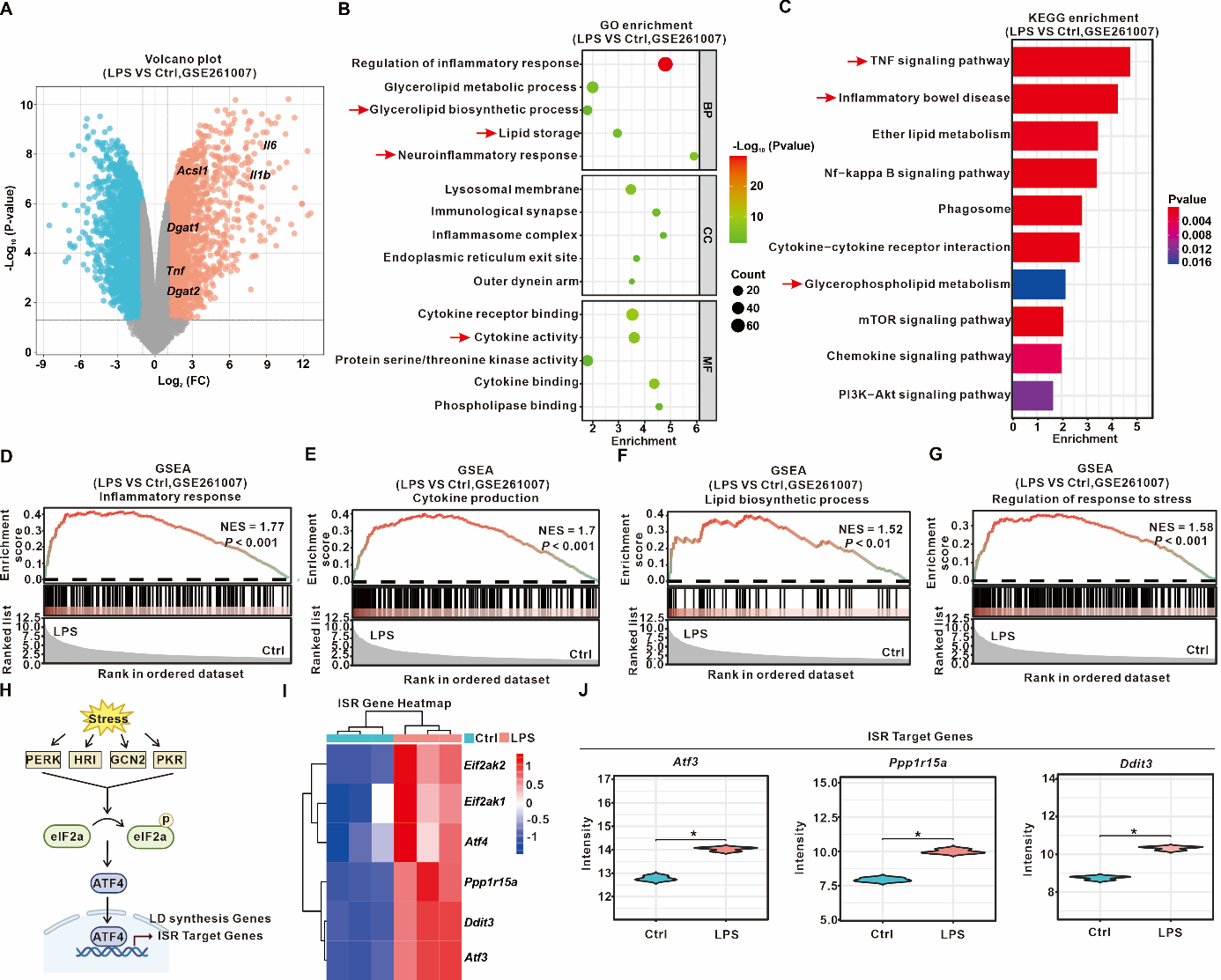
****Fig. S6.** **Gut-derived inflammatory stimulation enriches the ISR signaling pathway in microglia.** The GSE261007 dataset (RNA sequencing of primary microglia with or without LPS treatment; n = 3 per group) was analyzed. (A) Volcano plot of differentially expressed genes (DEGs); inflammation- and lipid droplet-synthesis genes highlighted. (B) GO enrichment of DEGs. (C) KEGG enrichment of DEGs. (D–G) GSEA of inflammatory response (D), cytokine production (E), lipid biosynthetic process (F), and regulation of response to stress (G) gene sets. (H) Schematic of ISR activation. (I) Heatmap of ISR-related genes. (J) Inter-group differences in canonical ISR target genes. * *P* < 0.05 versus control.

**
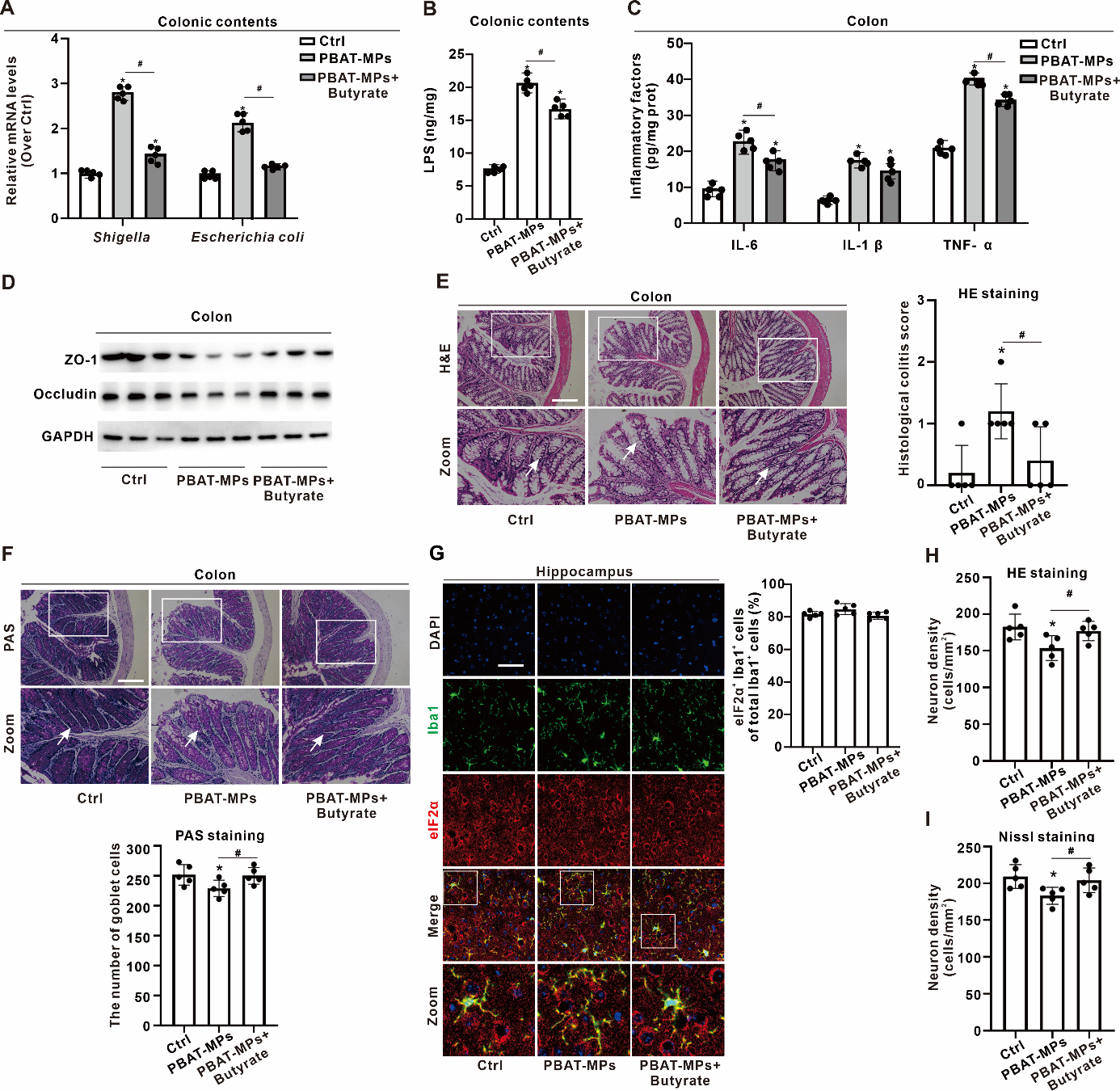
Fig. S7.** **Butyrate intervention alleviates partial colonic inflammation and injury.** C57BL/6 mice received daily oral gavage of PBAT-MPs (50 mg kg⁻¹) for 4 weeks, followed by intervention with or without butyrate for 2 weeks. n = 5 per group. (A) Relative mRNA levels of *Shigella* and *Escherichia coli* in colonic contents determined by qRT-PCR; n = 5. (B) LPS levels in colonic contents measured by ELISA; n = 5. (C) IL-6, IL-1β, and TNF-α levels in colon tissue measured by ELISA; n = 5. (D) ZO-1 and Occludin protein levels detected by WB; n = 3. (E) Representative H&E-stained images of colon tissue (left); scale bar, 100 μm. H&E-based colitis scores (right). (F) Representative PAS-stained images of colon tissue (left); scale bar, 100 μm. goblet cell counts (right). (G) Representative IF images of sections co-stained for Iba1 (green), eIF2a (red), and DAPI (blue) in hippocampal CA3 (left); scale bar, 50 μm. Quantification of eIF2a^+^Iba1^+^ cells as percentage of total Iba1^+^ cells (right). (H–I) Hippocampal neuronal density from H&E (H) and Nissl (I) staining. * *P* < 0.05 versus control; # *P* < 0.05 versus corresponding group.

**Supplementary tables**

**Table S1.** Primary antibodies employed for immunofluorescence (IF) in this study

| **Antibodies** | **Company** | **Dilution ratio** |
| --- | --- | --- |
| Alexa Fluor 488-labeled anti-rabbit/mouse | Beyotime | 1: 400 |
| Alexa Fluor 647-labeled anti-rabbit/mouse | Beyotime | 1: 400 |
| Anti-Iba1 | Abcam | 1: 200 |
| Anti-GFAP | Proteintech | 1: 400 |
| Anti-SOX10 | Abcam | 1: 200 |
| Anti-Plin2 | ABclonal | 1: 200 |
| Anti-TUJ1 | Proteintech | 1: 200 |
| Anti-eIF2α | Santa Cruz | 1: 200 |
| Anti-p-eIF2α | Proteintech | 1: 200 |
| Anti-ATF4 | ABclonal | 1: 200 |

**Table S2.** Primary antibodies employed for Western blotting (WB) in this study

| **Antibodies** | **Company** | **Dilution ratio** |
| --- | --- | --- |
| Anti-GAPDH | Beyotime | 1: 5000 |
| Anti-ZO-1 | Beyotime | 1: 1000 |
| Anti-Occludin | Beyotime | 1: 1000 |
| Anti-eIF2α | Santa Cruz | 1: 1000 |
| Anti-p-eIF2α | Proteintech | 1: 1000 |
| Anti-ATF4 | ABclonal | 1: 1000 |
| Anti-CHOP | Proteintech | 1: 1000 |
| Anti-puromycin | ABclonal | 1: 2000 |
| Anti-ACSL1 | ABclonal | 1: 1000 |
| Anti-DGAT1 | ABclonal | 1: 1000 |
| Anti-DGAT2 | ABclonal | 1: 1000 |
| Anti-Rptor | Proteintech | 1: 1000 |
| Anti-mTOR | Proteintech | 1: 1000 |
| Anti-p-mTOR | Proteintech | 1: 1000 |
| Anti-p-S6K1 | ABclonal | 1: 1000 |
| Anti-S6K1 | ABclonal | 1: 1000 |

**Table S3.** Primer sequences employed for qRT-PCR in this study

| **Species** | **Gene and primer name** | **Primer sequence** |
| --- | --- | --- |
| Human | *ACTB*-FP | 5'-CATGTACGTTGCTATCCAGGC-3' |
|  | *ACTB*-RP | 5'-CTCCTTAATGTCACGCACGAT-3' |
|  | *ATF3*-FP | 5'-CCTCTGCGCTGGAATCAGTC-3' |
|  | *ATF3*-RP | 5'-TTCTTTCTCGTCGCCTCTTTTT-3' |
|  | *PPP1R15A*-FP | 5'-ATGATGGCATGTATGGTGAGC-3' |
|  | *PPP1R15A*-RP | 5'-AACCTTGCAGTGTCCTTATCAG-3' |
|  | *DDIT3*-FP | 5'-GGAAACAGAGTGGTCATTCCC-3' |
|  | *DDIT3*-RP | 5'-CTGCTTGAGCCGTTCATTCTC-3' |
|  | *DGAT1*-FP | 5'-TATTGCGGCCAATGTCTTTGC-3' |
|  | *DGAT1*-RP | 5'-CACTGGAGTGATAGACTCAACCA-3' |
|  | *DGAT2*-FP | 5'-GAATGGGAGTGGCAATGCTAT-3' |
|  | *DGAT2-*RP | 5'-CCTCGAAGATCACCTGCTTGT-3' |
|  | *ACSL1-*FP | 5'-CTTATGGGCTTCGGAGCTTTT-3' |
|  | *ACSL1*-RP | 5'-CAAGTAGTGCGGATCTTCGTG-3' |
| Mouse | *Actb-*FP | 5'-GGCTGTATTCCCCTCCATCG-3' |
|  | *Actb-*RP | 5'-CCAGTTGGTAACAATGCCATGT-3' |
|  | *Atf3-*FP | 5'-TTTGCTAACCTGACACCCTTTG-3' |
|  | *Atf3*-RP | 5'-AGAGGACATCCGATGGCAGA-3' |
|  | *Ppp1r15a-*FP | 5'-GAGGGACGCCCACAACTTC-3' |
|  | *Ppp1r15a-*RP | 5'-GAGGGAGGAGGTTACCAGAGA-3' |
|  | *Ddit3-*FP | 5'-CTCGCTCTCCAGATTCCAGTC-3' |
|  | *Ddit3-*RP | 5'-CTTCATGCGTTGCTTCCCA-3' |

| Microbiota | *Shigella-*FP | 5'-CCTTGACCGCCTTTCCGATAC-3' |
| --- | --- | --- |
|  | *Shigella-*RP | 5'-CAGCCACCCTCTGAGAGTACTC-3' |
|  | *Escherichia coli-*FP | 5'-GTCACGCCGTATGTTATTG-3' |
|  | *Escherichia coli-*RP | 5'-CCAAAGCCAGTAAAGTAGAAC-3' |
|  | *Universal primers-*FP | 5'-ACTACGTGCCAGCAGCC-3' |
|  | *Universal primers-*RP | 5'-GGACTACCAGGGTATCTAATC-3' |
